# Sulfatase 2 inhibition sensitizes triple-negative breast cancer cells to paclitaxel through augmentation of extracellular ATP

**DOI:** 10.1080/15384047.2025.2483989

**Published:** 2025-03-26

**Authors:** Jasmine M. Manouchehri, Jharna Datta, Lynn M. Marcho, Daniel Stover, Ramesh K. Ganju, Bhuvaneswari Ramaswamy, William E. Carson, Arjun Mittra, Xiaoli Zhang, Patrick M. Schnell, Yu Yue, Mark P. Rubinstein, Mathew A. Cherian

**Affiliations:** a Comprehensive Cancer Center, The Ohio State University, Columbus, OH, USA; b College of Nursing, University of South Florida, Tampa, FL, USA; c College of Public Health, The Ohio State University, Columbus, OH, USA

**Keywords:** Chemotherapy, breast cancer, sulfatases, ATP, purinergic signaling, heparan sodium sulfate

## Abstract

The highest incidence and cancer-related mortality rate among women worldwide is due to breast cancer. Triple-negative breast cancers (TNBC) are associated with more inferior outcomes than other breast cancers because of their progressive nature and the deficit in available therapies. Therefore, there is a need for new therapeutic approaches. Our lab determined that chemotherapy induces the release of extracellular adenosine triphosphate (eATP), and, hence, augments TNBC cells’ response to chemotherapy. Despite this, eATP concentrations are restricted by a variety of extracellular ATPases. We propose that, as an ATPase inhibitor, heparan sulfate (HS) would augment eATP concentrations and TNBC vulnerability induced by chemotherapy. Sulfatase 2 (SULF2) removes sulfate from HS, the functional group essential for ATPase inhibition. Consequently, we propose that TNBC cell death and eATP release induced by chemotherapy would be intensified by SULF2 inhibitors. We examined eATP and cell viability in paclitaxel-treated TNBC and nontumorigenic immortal mammary epithelial MCF-10A cells in the presence of OKN-007, a selective SULF2 inhibitor, and/or heparan sodium sulfate. Furthermore, sulfatase 1 (SULF1) and SULF2 protein expressions were ascertained. We found that the expression of SULF2 was greater in TNBC cell lines when compared to MCF-10A cells. The release of eATP and loss of TNBC cell viability induced by chemotherapy was enhanced by OKN-007. The co-treatment of chemotherapy and OKN-007 also attenuated cancer-initiating cells. This data implies that the combination of SULF2 inhibitors with chemotherapy augments eATP and decreases cell viability of TNBC greater than chemotherapy alone.

## Background

Breast cancer diagnoses impacted 2,261,419 women in 2020.^1^ Among women, this form of cancer has the highest mortality and incidence rates.^2^ The mortality rate is largely due to the most aggressive form of breast cancer, triple-negative breast cancer (TNBC). Due to a lack of effective targeted therapies, chemotherapy is still the most efficacious treatment for TNBC, but a major downside is an inability to eradicate metastatic disease despite transient responses.^3^ Moreover, immunotherapy is only effective in a small subset of patients. Hence, there is a critical need for innovative therapeutic strategies.

The concentration of extracellular adenosine triphosphate (eATP) in tissues is between 0–10 nanomolar (nM) under physiological conditions while the concentration of adenosine triphosphate (ATP) intracellularly ranges from 3–10 millimolar (mM), a more than 10^6^-fold difference.^4^ However, this minute eATP quantity fulfills a critical role as a signaling molecule through cell surface purinergic receptors. Moreover, there is a marked increase in the concentration of eATP in the tumor microenvironment (TME), which can reach the micromolar range.^5–7^ Our published study showed that eATP is toxic to TNBC cells in the high micromolar range but not in nontumorigenic immortal mammary epithelial MCF-10A cells.^8^ Thus, cancer cells “live” closer to the threshold for cytotoxicity. Furthermore, our published data showed that chemotherapy treatment results in augmentation of eATP concentration.^8^ However, eATP concentration is limited by several families of ecto-nucleotidases, including ecto-nucleoside triphosphate diphosphohydrolases (ENTPD), 5’-nucleotidases (5’−NTs), ecto-nucleotide pyrophosphatases/phosphodiesterases (E-NPPase), and tissue nonspecific alkaline phosphatases (TNAP), with E-NTPD considered to be the key enzyme family responsible for ATP degradation and extracellular 5’-NT responsible for the catalytic conversion of AMP to adenosine and inorganic phosphate.^9^ However, we previously showed that inhibitors of each of these families of ecto-ATPases can augment chemotherapy-induced TNBC cell death and eATP release through P2RX4 and P2RX7 ion-coupled purinergic receptors. This data suggests that we need to inhibit all ecto-ATPases to maximize eATP release and TNBC cell death. The existence of several families of structurally diverse ecto-ATPases complicates the design of small-molecule inhibitors that can maximally suppress eATP degradation.

We noted that sulfated polysaccharides have been reported to inhibit multiple families of extracellular ATPases with nanomolar potency; the degree of sulfation, which imparts a negative charge to the molecule, is critical for this inhibition.^10^ We also noted that endogenous extracellular polysulfated polysaccharide heparan sulfate (HS) inhibits eATP degradation.^11^ Moreover, other publications show that the ecto-ATPase ecto-nucleotide pyrophosphatases/phosphodiesterase 1 (E-NPP1) binds to extracellular glycosaminoglycans, and its ATPase activity is competitively inhibited by these glycosaminoglycans, including heparin and HS.^12^

HS proteoglycans are ubiquitously expressed in the extracellular matrix and cell surface of animal cells. They can be broadly divided into three groups: transmembrane syndecans; glycosylphosphatidylinositol-linked glypicans; and extracellular matrix-associated perlecan, agrin, and collagen XVIII.^13^ The polysulfated polysaccharide HS is synthesized in the Golgi system and is composed of disaccharide units that are negatively charged and unbranched with sulfation of the 3-O, 6-O, or N sites of glucosamine along with the 6-O site of glucuronic/iduronic acid.^14–17^ HS plays a tumor suppressor role: loss of exostosin 1 or exostosin 2, proteins involved in the polymerization of HS, leads to hereditary multiple exostoses, a hereditary cancer syndrome associated with an elevated risk of chondrosarcomas and osteosarcomas.^18–21^ Sulfatase 1 (SULF1) and sulfatase 2 (SULF2) are extracellular HS 6-O-endosulfatases that hydrolyze the 6-O-sulfate groups on glucosamine residues in HS.^14,22–25^ SULF2 is highly expressed in a variety of cancers including breast cancer, while SULF1 is not.^15,22,25–27^ SULF2 has been shown to enhance tumor initiation and progression of a variety of cancers including breast (Supplemental Figure S1).^24,26,28^ When SULF2 was overexpressed in the breast cancer cell line MDA-MB 231, there was an increase in breast cancer cell growth.^24^ In contrast to SULF2, SULF1 is a tumor suppressor, possibly due to its negative regulation of fibroblast growth factor signaling.^22,29^

Both SULF1 and SULF2 are specific for 6-O sulfates of sulfated glucosamine residues of HS. However, SULF1 is selective for 6-O sulfates in the context of the trisulfated disaccharide sequence, 2-O sulfated uronic acid—2N, 6-O sulfated glucosamine in the “S” domains of HS.^30^ These are the regions of HS molecules where the majority of 2N sulfated glucosamine residues lie in contiguous sequences in contradistinction to “NA/NS” or transition domains of HS where N-acetylated glucosamine alternates with N-sulfated glucosamine, which are not substrates for SULF1.^30^ It is the 2-O sulfated Iduronic acid—2N, 6-O sulfated glucosamine disaccharide sequence in the “S” domains of HS that are selective docking sites for interactions between HS and proteins such as Noggin 1.^30^ Other publications also suggest some degree of difference between SULF1 and SULF2 regarding selectivity for S domains.^13^ These differences may explain why SULF1 has been reported to be a tumor suppressor in certain contexts while SULF2 is exclusively reported to be an oncogene.

**Figure 1.**
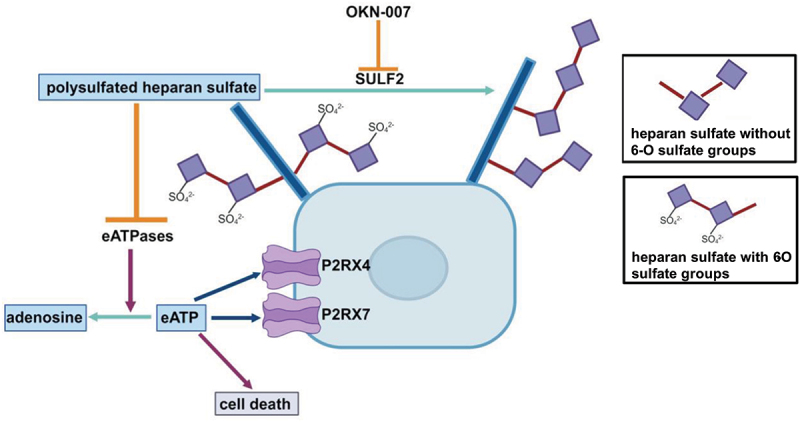
Schematic displaying our proposed model for heparan sulfate’s impact on eATP. Our proposed model suggests that accumulation of polysulfated polysaccharide HS, due to the presence of the sulfatase inhibitor OKN-007, facilitates ATP accumulation in the extracellular environment of paclitaxel-treated TNBC cells by preventing the breakdown of eATP by eATPases, which can lead to exacerbation of chemotherapy-induced cell death. This schematic was prepared using Biorender software (RRID:SCR_018361).

OKN-007 is a potent sulfatase inhibitor that has been demonstrated to decrease SULF2 activity.^31^ Moreover, OKN-007 is already in phase Ib clinical trials for glioblastoma multiforme (NCT03587038). Hence, we hypothesized that SULF2 inhibitors would accentuate the fully sulfated form of HS in the TME, an endogenous inhibitor of ecto-ATPases, thus enhancing TNBC cell death by augmenting eATP concentrations in the microenvironment of chemotherapy-treated cells (Figure 1).

## Methods

### Cell culture, drugs, and chemicals

Breast cancer cell lines MDA-MB 231 (ATCC HTB-26, RRID: CVCL_0062), MDA-MB 468 (ATCC HTB-132, RRID: CVCL_0419), Hs 578t (ATCC HTB-126, RRID: CVCL_0332), HEK-293T ATCC CRL-3216, RRID: CVCL_0063), T47D (ATCC HTB-13, RRID:CVCL-0553), MCF-7 (ATCC HTB-22 RRID:CVCL-0031), ZR-75-1 (ATCC CRL-1500 RRID:CVCL-0588) were maintained in DMEM (Corning; Cat# MT10013CV) and supplemented with 10% FBS (Gibco; Cat# A5256801), 1% MEM non-essential amino acids (Gibco; Cat# 11140050), 1 mm sodium pyruvate (Gibco; 11360070), 1mm Glutamax (Gibco; Cat# 35050061) and antimicrobial agents (100 units/ml Penicillin, 100 µg/ml streptomycin, and 0.25 µg/ml amphotericin B) (Gibco; Cat# 15140122). Non-tumorigenic immortalized mammary epithelial MCF-10A cells (ATCC Cat# CRL-10317, RRID:CVCL_0598) were maintained in DMEM/F12 (Gibco; Cat# 11320082) supplemented with 5% horse serum (Gibco; Cat# 26050088), hydrocortisone (Sigma; Cat# H0888-1 G), epidermal growth factor (Sigma; Cat# SRP3027), cholera toxin (Sigma; Cat# C8052), insulin (Sigma; Cat# 91077C) and antimicrobial agents. The cell lines were authenticated and maintained at 37°C, 5% CO_2_ and 95% relative humidity as described previously.^8^

The following drugs and chemicals were used: ATP (Sigma), dimethyl sulfoxide/DMSO (Sigma), paclitaxel (Calbiochem), OKN-007 (formally known as NXY-059) (Selleck Chemical), A438079 (Tocris), 5-BDBD (Tocris), heparan sodium sulfate (Sigma) and doxorubicin hydrochloride (Fisher). Heparan sodium sulfate was dissolved in nuclease-free water (Invitrogen); paclitaxel, OKN-007, A438079, 5-BDBD and doxorubicin hydrochloride were dissolved in dimethyl sulfoxide (DMSO) (Sigma). Table 1 shows the drugs’ concentrations and functions; we optimized the drug concentrations that were applied for the different assays by using previously described drug concentrations as starting points.^31–35^ Cells were treated at the designated concentrations.

**Table 1.** Drug concentrations and functions.

Drug	Concentration(s)	Function	Concentration reference
paclitaxel	50 and 100 µM	Chemotherapeutic agent	^32^
OKN-007	20 µM	Sulfatase inhibitor	^31^
A437809	20 µM	P2RX7 inhibitor	^33^
5-BDBD	20 µM	P2RX4 inhibitor	^34^
heparan sodium sulfate	50 µM	developmental processes, angiogenesis, and tumor metastasis	^35^
doxorubicin	10 and 25 µM	Chemotherapeutic agent	^36^

### Measurement of ecto-ATPase inhibition by heparan sulfate

ATP was combined with recombinant proteins (enzymes): ENPP1, tissue nonspecific alkaline phosphatase (TNAP), and ecto-nucleoside triphosphate diphosphohydrolases 1 (CD39/ENTPD1) (R&D Systems) in the presence or absence of heparan sodium sulfate. The amount of eATP was analyzed according to the protocol described by the manufacturer of ATPlite 1-step Luminescence Assay System (PerkinElmer, Cat# 6016736) either at 0 hours (eATP concentrations were determined immediately) or after 48 hours of incubation at 37°C. Luminescence readings were obtained from a Biotek Synergy HT plate reader. As a control, enzymes were boiled, and the subsequent experiments were carried out in the same fashion.

### Western blot analysis of expression of sulfatases

An equal number of individual cell lines: TNBC MDA-MB 231, Hs 578t, MDA-MB 468 cells, estrogen and progesterone receptor positive (ER+/PR+) T47D, MCF-7, ZR-75-1 cells and MCF-10A cells were seeded and cultured for 48 hours to 70%-80% confluency. Cell supernatants were collected. Total cell lysates were prepared, protein quantification was performed; and proteins were denatured, separated, and transferred as previously described.^8^ For immunoblot of SULF1 and SULF2 on cell lysates, 100 µg of protein was loaded onto gels. For immunoblot of SULF1 and SULF2 on cell supernatants, a volume that was normalized to the total mass of protein measured in the corresponding lysate was loaded onto the gels. The membranes were blocked with 5% nonfat milk at room temperature for an hour and incubated overnight at 4°C with a primary antibody: SULF1 (1:200 dilution; Novus Biologicals, Cat# NBP231584, RRID: AB_2916043) and SULF2 (1:500 dilution; Abcam, Cat# ab232835, RRID: AB_2916044) diluted in 5% nonfat milk. The membranes were washed and developed as described previously.^8^ β-actin (Genetex, Cat# GTX109639; RRID:AB_1949572) was used as a loading control. Densitometry was performed on Licor Image Studio (RRID: SCR_015795). All experiments were repeated multiple times.

### ELISAs

TNBC cell lines, MDA-MB 231, Hs 578t and MDA-MB 468, ER+/PR+ cell lines, T47D, MCF-7 and ZR-75-1, and MCF-10A cell line were grown for 48 hours to 70–80% confluency, and supernatants were collected. The concentrations of SULF1 and SULF2 (Lifespan Biosciences, Cat# LS-F66757-1 and LS-F35926-1) were measured in the supernatants normalized to the total mass of protein of cell lysates of the examined cell lines via enzyme-linked immunoassay (ELISA) analysis according to the manufacturer’s directions.

### Immunohistochemistry of sulfatase 2

AMSBIO BR1202B breast cancer tissue array (120-core array: 82 TNBC cores, 20 estrogen receptor/progesterone receptor positive (ER+/PR+) cores, 14 human epidermal growth factor receptor 2 positive (HER2+) cores and 4 necrotic cores) was sectioned at 5 µm and air-dried overnight on Fisher Superfrost Plus slides. Also, normal breast tissue or ductal carcinoma in situ (DCIS) was prepared on PERMAFLEX plus slides. All stainings were applied to the Leica Bond RX automated stainer (Leica Microsystems) and applying a standard operating procedure with a fully automated workflow that were carried out at Histowiz, Inc. (Brooklyn, NY). Normal breast and DCIS samples were processed, embedded in paraffin, and sectioned at 4 μm. The slides were dewaxed using xylene and alcohol-based dewaxing solutions. Immunohistochemistry: Epitope retrieval was carried out by heat-induced epitope retrieval (HIER) of the formalin-fixed, paraffin-embedded tissue using citrate-based pH 6 solution (Leica Microsystems, Cat#AR9961) for 10 minutes at 95°C. The tissues were first incubated with a peroxide block buffer (Leica Microsystems, Cat# RE7101-CE), followed by incubation with the rabbit anti-SULF2 antibody (Abcam, Cat# ab232835, RRID: AB_2916044) at 1:50 dilution for 30 minutes, followed by DAB rabbit secondary reagents: polymer, DAB refine, and hematoxylin (Bond Polymer Refine Detection Kit, Leica Microsystems, Cat# DS9800) according to the manufacturer’s protocol. The slides were dried, coverslipped (TissueTek-Prisma Coverslipper), and visualized using a Leica Aperio AT2 slide scanner (Leica Microsystems) at 40 ×.

Quantitation of protein expression: Automated analysis of protein expression was performed at Histowiz, Inc. For the analysis of the tissue microarray (TMA), the Halo TMA module was applied to identify and extract the individual TMA cores by means of constructing a grid over the TMA. All subsequent analysis steps were the same for the 3 slides with DCIS tissue and 2 slides with normal breast tissue and the TMA slide. In the first part of the analysis, the tumor area was determined by training a random forest classifier algorithm to separate viable tumor tissue from any surrounding stroma and necrosis areas. Once the tumor area was determined, the analysis then proceeded to classify positive and negative cells based on SULF2 staining within the defined tumor area on each slide and each core from the TMA slide. Positive and negative cells were distinguished using the Halo Multiplex IHC algorithm v3.4.1 by first defining the settings for the hematoxylin counterstain, followed by setting thresholds to detect the SULF2 stain positivity of weak, moderate, and strong (Halo threshold settings 0.11, 0.35, 0.45). An H-score was then generated using the formula (percentage cells with 1+ staining x 1) + (percentage cells with 2+ staining x 2) + (percentage cells with 3+ staining x 3) following the convention below: weak positive (1+), moderate positive (2+), and strong positive (3+).

### Cell viability and eATP assays

TNBC MDA-MB 231, Hs 578t, MDA-MB 468 cells, and MCF-10A cells were plated as previously described and treated with paclitaxel (paclitaxel alone referred to as “vehicle addition”), heparan sodium sulfate (50 µM), OKN-007 (20 µM), A438709 (20 µM), 5-BDBD (20 µM), or different combinations of these drugs. Cells were treated with OKN-007 and heparan sodium sulfate for 48 hours and with paclitaxel, A438709, or 5-BDBD for the final 6 hours of the 48-hour time course (we treated cells with paclitaxel for 6 hours to replicate exposure times in patients); cell viability was assessed by applying the PrestoBlue™ HS cell viability reagent (Invitrogen, Cat# P50201) following the manufacturer’s instructions.^8^ ATP was assessed in supernatants as described above. Fluorescence readings (excitation and emission ranges: 540–570 nm and 580–610 nm) were assessed using a Biotek Synergy HT plate reader.

### Sulfatase activity assay

The sulfatase inhibitor OKN-007 (20 µM) or vehicle control was added to recombinant sulfatase enzyme (0, 2, 4 µL) with sulfatase substrate (p-nitrocatechol sulfate) diluted in the sulfatase assay buffer, and sulfatase activity was determined by comparison with the p-nitrocatechol standard dilution curve. The p-nitrocatechol standard was prepared according to the manufacturer’s protocol for the sulfatase activity assay kit (Sigma, Cat# MAK276-1KT). Absorbance readings at 515 nm were obtained from a Biotek Synergy HT plate reader.

### Flow cytometry for cancer-initiating cells

TNBC MDA-MB 231, Hs 578t, and MDA-MB 468 cells were collected and stained following the protocol for the Aldefluor Kit (STEMCELL, Cat# 01700). Cells were washed and stained with CD24-PE (eBioscience, Cat# 12-0247-42, RRID: AB_1548678), CD44-APC (eBioscience, Cat# 17-0441-82, RRID: AB_469390), and LIVE/DEAD™ Fixable Near-IR Dead Cell Stain Kit (Invitrogen, Cat# L10119) for 30 minutes at 4°C. Cells were washed and resuspended in the Aldefluor Buffer provided in the Aldefluor kit. Flow cytometry analysis was performed on BD fluorescence-activated cell sorting (FACS) Fortessa using the FITC (ALDH), PE (CD24), APC (CD44), and AF750 (Live/Dead) channels and applying the Flowjo software (RRID: SCR_008520).

### Tumorsphere formation efficiency assay

TNBC MDA-MB 231, Hs 578t, and MDA-MB 468 cell lines were grown and treated with paclitaxel, OKN-007, and/or heparan sodium sulfate as described above in the “Cell viability and eATP assays” section. Cells were trypsinized, washed, resuspended in 3D Tumorsphere Medium XF (Sigma), and plated at 10 viable cells per well after (45 µM) filtration on low-attachment, round-bottom 96-well plates. Cells were grown for 7 days, and tumorspheres were counted for each different condition using the Etaluma™ Lumascope 620.

### Statistical and bioinformatics analyses

When displayed to be statistically significant, a post hoc Dunn’s test was done to determine *p* values. *p* values were adjusted to account for multiple comparisons and an alpha level of 0.05 was used for all tests for IHC analysis. The software GraphPad Prism version 10.0.2 (RRID: SCR_002798) was applied for all tests The student’s t-test was applied to the applicable assays to determine significance, including for western blot densitometry. One-way ANOVA with Tukey’s honestly significant difference (HSD) was calculated to determine significance for cell viability, eATP assays and tumorsphere formation efficiency assays.

Bliss independence models were obtained from estimated mean viabilities under paclitaxel and OKN-007 alone via the formula Log_Viability (Bliss) = Log_Viability(paclitaxel) + Log_Viability (OKN-007). Interaction at each dose was quantified as the ratio of the predicted viability under the Bliss independence model over the estimated viability under the tested paclitaxel + OKN-007 combination, with ratios > 1 indicating synergy.

## Results

### Effect of heparan sulfate on ecto-ATPase activity

**Figure 2.**
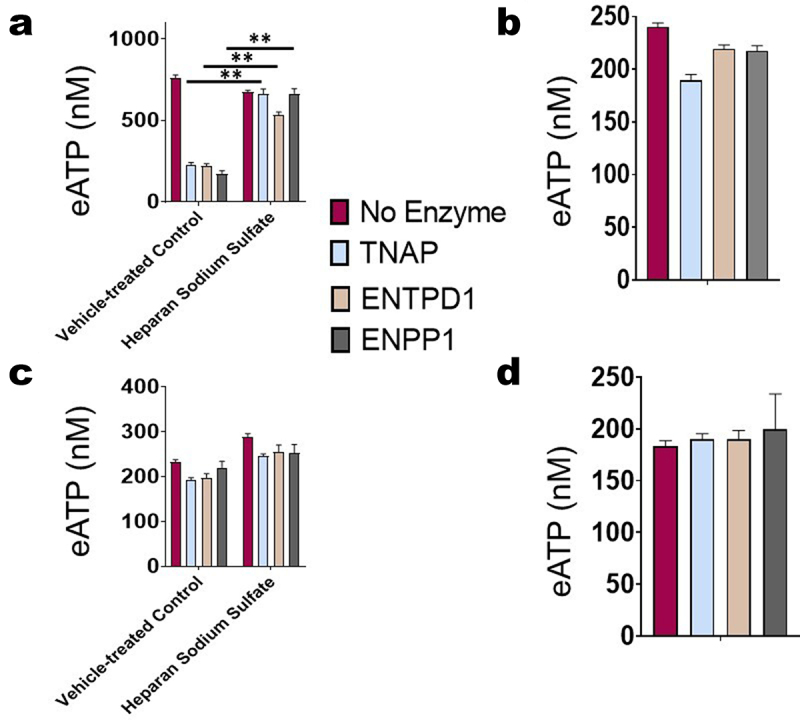
Heparan sulfate’s influence on eAtpases’ activity. ATP (500 µm) was incubated with vehicle or 100 units of one member of each of the families of extracellular ATPases, tissue non-specific alkaline phosphatase (TNAP), ENTPD1, and ENPP1, in the presence or absence of heparan sodium sulfate (50 µm) for (a) 48 hours at 37°C or (b) 0 hours. For (c) and (d), enzymes were boiled before incubation, and eATP was measured after (c) 48 hours at 37°C or (d) 0 hours. ATP concentrations were measured using a luciferase-based assay. HS blocked eATP degradation by all three families of enzymes. The standard deviation was calculated from three independent experiments performed in triplicate. The student’s t-test was performed to determine significance, with **indicating *p* value <0.01 for the comparison of enzyme-treated ATP vs. enzyme and heparan sodium sulfate.

We first sought to determine if polysulfated HS prevents eATP degradation by different families of ecto-ATPases: tissue nonspecific alkaline phosphatase (TNAP), ecto-nucleoside triphosphate diphosphohydrolases 1 (ENTPD1), and ecto-nucleotide pyrophosphatase/phosphodiesterase (ENPP1). We incubated ATP (500 µM) with vehicle or 100 units of one member of each of the families of ecto-ATPase enzymes in the presence or absence of heparan sodium sulfate (50 µM) for 48 hours or 0 hours (ATP concentrations determined immediately) at 37°C (Figures 2a,b). In the absence of heparan sodium sulfate, all three enzymes significantly decreased the concentration of ATP after 48 hours; however, in the presence of heparan sodium sulfate, eATP concentrations were not suppressed. Additionally, as a control, we used boiled enzymes added to ATP in the presence or absence of heparan sodium sulfate, and the eATP concentrations did not change, demonstrating that the reduction in ATP observed with the addition of enzymes is specifically due to their catalytic activity (Figures 2c,d). We also did not observe any change in ATP concentration when its level was measured at 0 hours (immediately) after the addition of enzyme. This result indicates that HS does not positively or negatively interfere with the ATP assay. These results demonstrate that HS inhibits the ATPase activity of each of the three major families of ecto-ATPases.

### Analysis of expression of sulfatases in breast cancer

#### Expression of sulfatases in breast cancer cell lines and mammary epithelial cells by Western blot and ELISA

**Figure 3.**
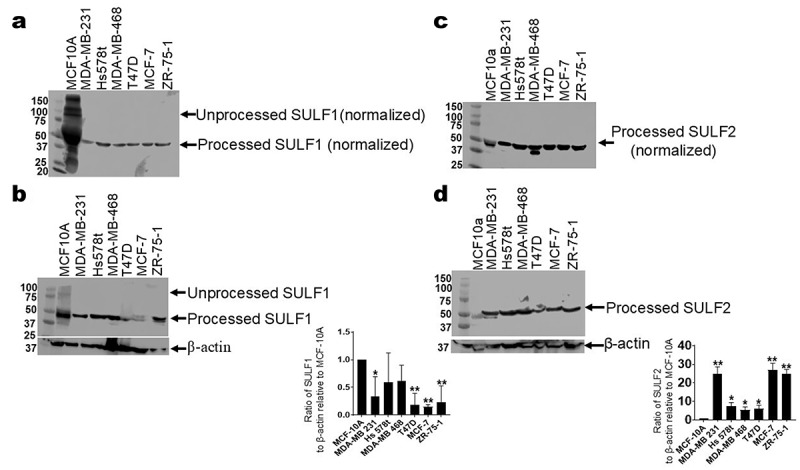
Immunoblot analysis of SULF1 and SULF2 expression in immortal mammary epithelial cells, TNBC cell lines and ER+/PR+ cell lines. For the Western blot analysis of SULF1, (a) Adjusted volume of cell supernatants inversely proportionate to the protein concentration in the corresponding cell lysate of MCF-10A cells, TNBC cell lines—MDA-MB 231, Hs 578t, and MDA-MB 468 cells and ER+/PR+ cell lines-T47D, MCF-7 and ZR-75-1 were loaded for polyacrylamide gel electrophoresis (PAGE) and probed with a SULF1-specific antibody. (b) Equal amounts of cell lysate from each cell line were probed with a SULF1-specific antibody. β-actin was used as a loading control. Western blotting was performed multiple times. For the Western blot analysis of SULF2, (c) Adjusted volume of cell supernatants inversely proportionate to the protein concentration in the corresponding cell lysate of MCF-10A cells, TNBC cell lines—MDA-MB 231, Hs 578t, and MDA-MB 468 cells— and and ER+/PR+ cell lines-T47D, MCF-7 and ZR-75-1 were subjected to PAGE and probed with a human SULF2-specific antibody. (d) Equal amounts of cell lysate from each cell line were probed with a SULF2-specific antibody. β-actin was used as a loading control. Western blotting was performed multiple times. The densitometric analyses of the bands for (b) SULF1 and (d) SULF2 were performed using image studio software (LI-COR Inc.). The student’s t-test was performed to determine significance with * representing *p* < 0.05 and ** representing *p* < 0.01 when comparing expression in MCF-10A cells to expression in TNBC cell lines and ER+/PR+ cell lines. Cropped blots are shown in the figure and full-length blots are presented in supplementary figures 2–5.

To compare the level of expression of SULF1 and SULF2 in TNBC cell lines to that in control MCF-10A cell line, we performed Western blot analysis on TNBC MDA-MB 231, Hs 578t, and MDA-MB 468 cell lines and MCF-10A cells, probing for SULF1 and SULF2 with β-actin as the internal loading control (Figure 3; Supplemental Figures S2–S5; Table 2). We probed for SULF1 protein and determined that the TNBC cell lines express less SULF1 extracellularly in comparison to MCF-10A cells (Figure 3a), but Hs 578t was the only TNBC cell line to express more intracellular SULF1 protein in comparison to MCF-10A cells (Figure 3b). We also found that TNBC cell lines expressed markedly higher levels of SULF2 protein extracellularly (Figure 3c) as well as intracellularly (Figure 3d) when compared to MCF-10A cells, as assessed by semi-quantitative densitometry.

**Table 2.** SULF1 and SULF2 expression.

TNBCs’ expression relative to MCF-10A cells	SULF1	SULF2
Extracellular	Less	More
Intracellular	Less	More

**Figure 4.**
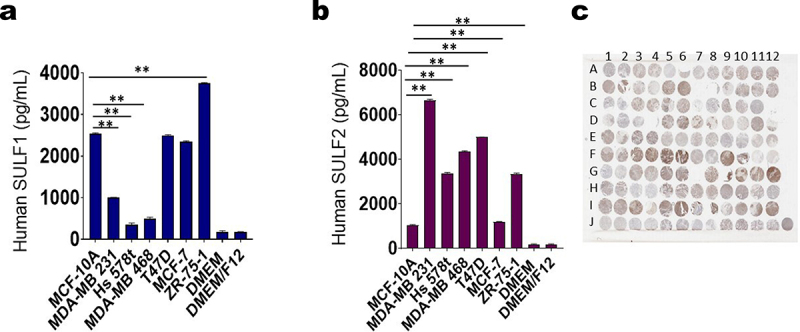
ELISA analysis of sulfatases and SULF2 immunohistochemistry. ELISAs were performed to examine the basal levels of (a) SULF1 and (b) SULF2 in adjusted cell supernatants inversely proportionate to the protein concentration in the corresponding cell lysate of MCF-10A cell line, TNBC MDA-MB 231, Hs 578t and MDA-MB 468 cell lines and ER+/PR+ T47D, MCF-7 and ZR-75-1 cell lines. MCF-10A expressed more SULF1 in comparison to the TNBCs but similar expression levels were observed in ER+/PR+ T47D and MCF-7 cells. However, the ER+/PR+ ZR-75-1 cell line expressed the most SULF1. Meanwhile, TNBCs and ER+/PR+ T47D and ZR-75-1 cells expressed more SULF2 in comparison to MCF-10A cells. Standard deviation was calculated from three independent experiments performed in triplicate. The student’s t-test was performed to determine significance with *representing *p* < 0.05 and **representing *p* < 0.01 comparing the protein expression MCF-10A to the protein expressions of TNBC cell lines. (b) The AMSBIO BR1202B breast cancer tissue array (120 cores with 82 TNBC cores, 20 ER+/PR+ cores, 14 HER2+ cores, and 4 necrotic cores; key can be found in supplemental Figure S6C) was stained with SULF2.

We observed through ELISAs that TNBC cell lines expressed less SULF1 in their supernatants in comparison to control MCF-10A cells aligning with the Western blot analysis. Meanwhile, ER+/PR+ ZR-75-1 cell line expressed more SULF1 in comparison to MCF-10A, but other ER+/PR+ cell lines had similar expressions to MCF-10A cells (Figure 4a). We also found that TNBC and some ER+/PR+ cell lines expressed significantly more SULF2 in their supernatants in comparison to MCF-10A cells aligning with the Western blot analysis (Figure 4b). Therefore, targeting SULF2 expression could be an effective way to overcome chemotherapeutic resistance in TNBCs.

### Measurement of SULF2 expression in human breast cancer samples by immunohistochemistry

**Figure 5.**
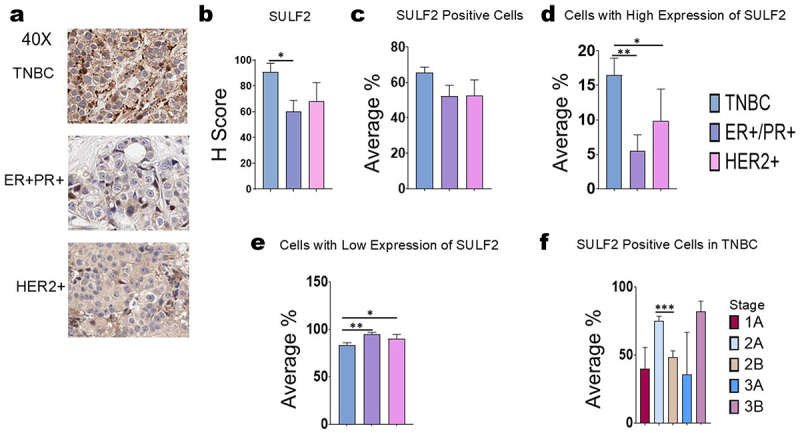
Statistical analysis for SULF2 immunohistochemistry. For the breast cancer tissue array and slides stained for heparanase: TNBC (*n*=75), ER+/PR+ (*n*=18), HER2+ (*n*=14), (a) images were taken of SULF2-stained AMSBIO BR1202B breast cancer tissue array on an evos FL auto 2 microscope (40×). (b) Pairwise comparisons using Dunn’s test indicated that there was a significant difference in H-score between breast cancer sub-types TNBC and ER+/PR+ (*p* = 0.0392). There was no significant different between TNBC and HER2+. (c) The Kruskal-Wallis test indicated that there was no significant difference in the average percentages of cells that stained positively for SULF2 in tissue sections of TNBC, ER+/PR+ breast cancer, and HER2+ breast cancer. (d) Pairwise comparisons using Dunn’s test indicated that there was a significant difference between breast cancer sub-types TNBC and ER+/PR+ (*p* = 0.009), and TNBC and HER2+ (*p* = 0.0338), in the percentages of cells that stained moderately or strongly positive for SULF2. (e) The same Dunn’s test from (d) also indicated that there was a significant difference between cancer sub-types TNBC and ER+/PR+ (*p* = 0.0094), and TNBC and HER2+(*p* = 0.0338), in the percentages of cells that stained negative or weakly positive for SULF2. (f) Pairwise comparisons using Dunn’s test showed that there was a significant difference between TNBC breast cancer stages 2A and 2B (*p* = 0.0007) in the percentages of cells that stained positively for SULF2. No other differences among different TNBC stages were significant. Standard error of the mean was calculated. For statistical analysis, *representing *p* < 0.05, **representing *p* < 0.01 and ***representing *p* < 0.001.

An AMSBIO BR1202B breast cancer tissue array (120 cores with 82 TNBC cores, 20 ER+/PR+ cores, 14 HER2+ cores, and 4 necrotic cores) was stained with SULF2 (Figure 4c; Supplemental Table S1; Supplemental Figure S6). We focused on SULF2 because SULF1 has been reported to be a tumor suppressor.^24,37^ Statistical analysis was performed on the breast cancer tissue array stained with SULF2 antibody. The expression of SULF2 was compared between TNBCs, ER+/PR+, HER2+ breast cancer, normal breast tissue, and DCIS. Through statistical analysis, we found that there was a significant difference in H-score between breast cancer sub-types TNBC and ER+/PR+, but there was no significant difference in H-score between TNBC and HER2+ (Figure 5b). We also calculated no statistically significant difference in the average percentages of cells that stained positively for SULF2 in tissue sections of TNBC, ER+/PR+ breast cancer, and HER2+ breast cancer (Figure 5c). We determined that there was a statistically significant difference between breast cancer sub-types TNBC and ER+/PR+ and TNBC and HER2+, in the percentages of cells that stained moderately or strongly positive for SULF2 (Figure 5d). Additionally, we observed a statistically significant difference between breast cancer sub-types TNBC and ER+/PR+ and TNBC and HER2+ of the average percentages of cells that stained negative or weakly positive for SULF2 (Figure 5e). These results consistently show that TNBC express more SULF2 than other breast cancer sub-types. Furthermore, we found that there was a statistically significant difference between the percentages of cells that were positive for SULF2 in tissue sections of TNBC breast cancer stages 2A vs. 2B (Figure 5f). The different stages refer to tumor size, but the pattern between tumor size and SULF2 expression is not clear because stage 2B’s SULF2 expression is lower than the lesser tumor stage 2A’s SULF2 expression; whereas, the higher stage 3B had the highest SULF2 expression. Additional statistical analysis was carried out comparing the SULF2 expression among TNBCs, ER+/PR+, HER2+ breast cancer, normal breast tissue, and DCIS (Supplemental Figure S7). We also compared SULF2 among normal breast tissue, DCIS, and various grades of cancers (Supplemental Figure S8). Additionally, we compared SULF2 expression among different PR expression levels (Supplemental Figure S9). Furthermore, we observed that the higher percentage of Ki67 was associated with a higher expression of SULF2 (Supplemental Figure S10).

### Effect of sulfatase inhibitor effect on eATP and cell viability

**Figure 6.**
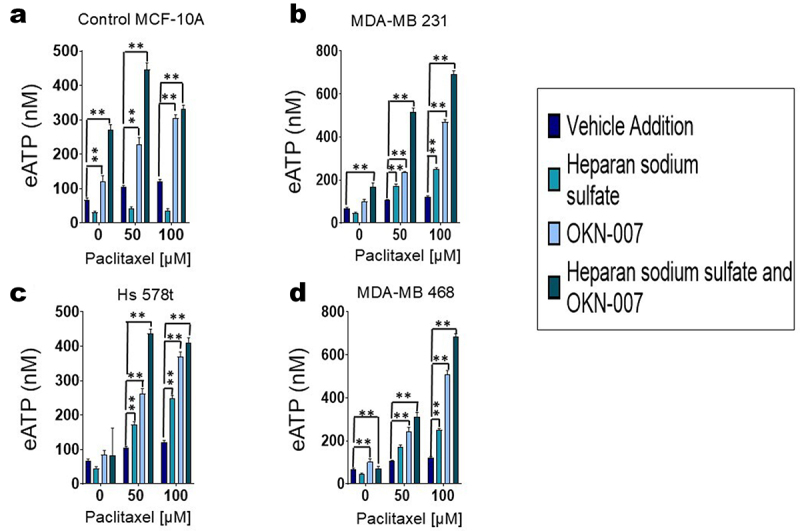
Effect of sulfatase inhibitor OKN-007, chemotherapeutic agent paclitaxel and their combination on extracellular ATP concentrations. Extracellular ATP concentrations were measured in the supernatants of treated (a) MCF-10A cells and triple-negative breast cancer cell lines (b) MDA-MB 231, (c) Hs 578t, and (d) MDA-MB 468 cells. The treatments: vehicle addition (paclitaxel, purple), heparan sodium sulfate (50 µm, teal) and OKN-007 (20 µm, light blue), or the combination of all 3 (dark blue-green); heparan sodium sulfate and OKN-007 were administered for 48 hours and paclitaxel was added for the final 6 hours to replicate exposure times to paclitaxel in patients. Standard deviation was calculated from three independent experiments performed in triplicate. One-way ANOVA with Tukey’s HSD was applied to ascertain significance. *represents *p* < 0.05 and **represents *p* < 0.01 when comparing vehicle addition to heparan sodium sulfate, OKN-007, or the combination regimen.

**Figure 7.**
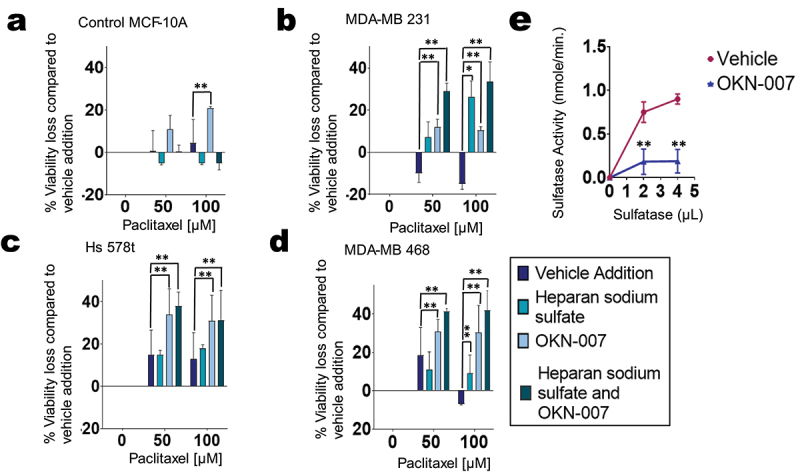
Effects of sulfatase inhibitor OKN-007 combined with chemotherapeutic agent paclitaxel on cell viability. Percentage loss of cell viability was measured in treated (a) MCF-10A cells and TNBC cell lines (b) MDA-MB 231, (c) Hs 578t, and (d) MDA-MB 468 cells. The treatments applied were vehicle addition (paclitaxel, purple), heparan sodium sulfate (50 µm, teal), and OKN-007 (20 µm, light blue) or the combination (dark blue-green); heparan sodium sulfate and OKN-007 were administered for 48 hours, and paclitaxel was added for the final 6 hours to replicate paclitaxel exposure times in patients. Standard deviation was calculated from three independent experiments performed in triplicate. One-way ANOVA with Tukey’s HSD was applied to ascertain significance. *represents *p* < 0.05 and **represents *p* < 0.01 when comparing vehicle addition to heparan sodium sulfate, OKN-007, or the combination. (e) A sulfatase activity assay was carried out. OKN-007 (20 µm) was added to the vehicle (sulfatase; 0, 2, 4 µl) and sulfatase substrate; sulfatase activity (desulfation of p-nitrocatechol sulfate to p-nitrocathecol) was determined using a standard dilution of p-nitrocatechol. There was a decrease in sulfatase activity when sulfatase and sulfatase substrate were exposed to OKN-007, confirming the antagonistic effect of this inhibitor on sulfatase. The standard deviation was calculated from three independent experiments performed in triplicate. The student’s t-test was performed to determine significance with *representing *p* < 0.05 and **representing *p* < 0.01, comparing the sulfatase activity between the vehicle (sulfatase) to OKN-007 and the vehicle.

We previously demonstrated that eATP augmentation by ecto-ATPase inhibitors increases chemotherapy-induced TNBC cell death.^8^ Given that polysulfated heparan is an ecto-ATPase inhibitor, we next determined the effect of combining the sulfatase inhibitor (OKN-007) with chemotherapy (paclitaxel) to ascertain its impact on the viability of TNBC MDA-MB 231, Hs 578t, and MDA-MB 468 cells in comparison to MCF-10A cells and its effects on eATP release. For these experiments, all the cell lines were treated with vehicle (paclitaxel alone), OKN-007 and/or heparan sodium sulfate for 48 hours and then paclitaxel or a corresponding vehicle was added to the medium for the final 6 hours. The 6-hour duration of paclitaxel exposure was used to simulate the short duration of systemic exposure in cancer patients (Figures 6 and 7).^38^ We measured the concentration of eATP in the supernatants of the chemotherapy-treated (paclitaxel) cells (Figure 6). Upon treatment with the combination of OKN-007 (20 µM) and paclitaxel (100 µM), we saw significant increases in eATP levels when compared to vehicle addition in MCF-10A cells as well as TNBC MDA-MB 231, Hs 578t, and MDA-MB 468. There was also a significant increase in eATP with the addition of heparan sodium sulfate to the combination of paclitaxel and OKN-007 when compared to the vehicle addition. Therefore, the sulfatase inhibitor OKN-007 significantly increased eATP release upon chemotherapy treatment and sensitized TNBC cell lines to chemotherapy treatment.

Under the same conditions, we checked cell viability. As the treatment with paclitaxel was for only 6 hours, we did not see changes in the viability of cells treated with paclitaxel alone (vehicle addition). However, in all 3 TNBC cell lines – MDA-MB 231, Hs 578t, and MDA-MB 468—when paclitaxel was combined with the sulfatase inhibitor OKN-007, there was a significant loss of cell viability when compared to paclitaxel alone (shown as mean percentage loss of viability compared to vehicle control) (Figure 7). Additionally, the loss of cell viability was further increased significantly when heparan sodium sulfate was added to the combination of paclitaxel and OKN-007 when compared to paclitaxel alone. However, there was no significant change in the viability of MCF-10A cells treated with paclitaxel, OKN-007, and heparan sodium sulfate compared to paclitaxel alone, suggesting that this effect may be selective for transformed cells.

### Effect of OKN-007 on sulfatase activity

We wanted to confirm that the SULF2 inhibitor OKN-007 inhibits sulfatase activity at the concentrations planned for our experiments. We utilized a sulfatase activity kit that can measure the desulfation of p-nitrocatechol sulfate by sulfatase to p-nitrocatechol. We added the SULF2 inhibitor OKN-007 (20 µM) to recombinant sulfatase (0, 2, 4 µL) and sulfatase substrate diluted in the sulfatase assay buffer, and sulfatase activity was determined through a calculated standard dilution of p-nitrocathecol (Figure 7e). We found there was significantly decreased sulfatase activity when sulfatase and sulfatase substrate were exposed to OKN-007. These results demonstrate that OKN-007 neutralizes sulfatase activity at the concentration utilized (20 µM). Thus, we were able to apply OKN-007 to our experiments to inhibit SULF2 to increase TNBCs’ response to chemotherapy.

### Effect of SULF2 inhibition on efficacy of doxorubicin

Additionally, we tested an additional chemotherapeutic agent doxorubicin in TNBC MDA-MB 231 cells in combination with OKN-007 and heparan sodium sulfate (Supplemental Figure S11). We found that combination of doxorubicin, OKN-007 and heparan sodium sulfate produced an increase in eATP and increase in the percent loss of cell viability in MDA-MB 231 cells.

### Effect of paclitaxel and extracellular ATP on the expression of sulfatase 2

We also treated TNBC MDA-MB 231, Hs 578t and MDA-MB 468 cells and MCF-10A cells with paclitaxel (100 µM) for 6 hours or ATP (500 µM) for 48 hours and were probed for SULF2 (Supplemental Figures S12 and S13). We did not observe any change in SULF2 expression in the presence of paclitaxel or ATP.

### Synergy of paclitaxel with SULF2 inhibitor analyzed with dose-response curves

We also obtained dose response graphs for treatments of increasing concentrations of paclitaxel and OKN-007 (Supplemental Figure S14). There was some synergy (<0.1–1.0) for some dose combinations (paclitaxel and OKN-007) for MDA-MB 231 cells while there were some additive drug dose combinations (paclitaxel and OKN-007) (1–1.2) for Hs 578t and MDA-MB 468 cells.

### Role of purinergic signaling in the augmentation of chemotherapy-induced TNBC cell death by SULF2 inhibitors

**Figure 8.**
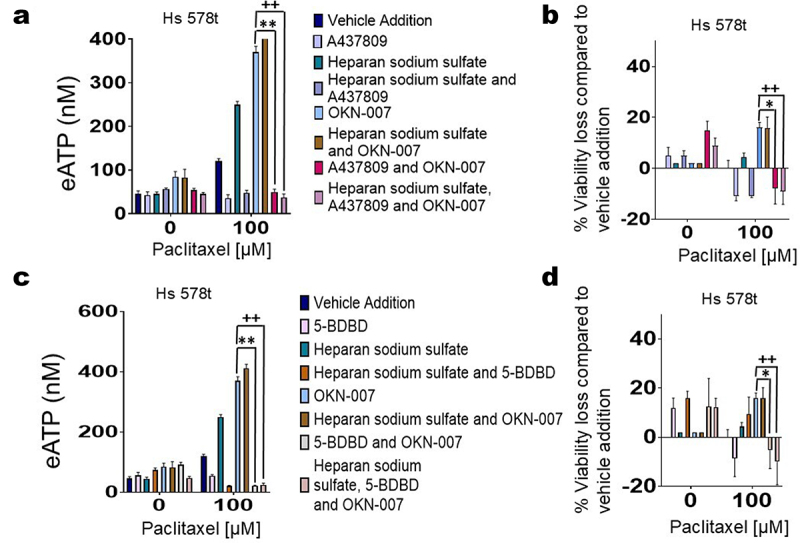
Reversal of sulfatase inhibitor’s effects by P2RX4 and P2RX7 inhibitors. For experiments corresponding to figures (a) and (b), Hs 578t cells were treated with OKN-007 (20 µm, 48 hours), paclitaxel (100 µm, the final 6 hours of the 48-hour time course to replicate exposure times in patients), heparan sodium sulfate (50 µm, 48 hours), the P2RX7 antagonist A437809 (20 µm, 6 hours), or a combination of the different drug agents. Standard deviation was calculated from three independent experiments performed in triplicate. One-way ANOVA with Tukey’s HSD was applied to ascertain significance. *represents *p* < 0.05 and **represents *p* < 0.01 when comparing OKN-007 to the combination of vehicle addition, OKN-007, and A437809; + represents *p* < 0.05 and ++ represents *p* < 0.01 when comparing OKN-007 to the combination of vehicle addition, OKN-007, A437809, and heparan sodium sulfate. For experiments corresponding to figures (c) and (d), Hs 578t cells were treated with OKN-007 (20 µm, 48 hours), paclitaxel (100 µm, final 6 hours of the 48-hour time course to replicate exposure times in patients), heparan sodium sulfate (50 µm, 48 hours), the P2RX4 antagonist 5-BDBD (20 µm, 6 hours), or combinations of these agents. Standard deviation was calculated from three independent experiments performed in triplicate. One-way ANOVA with Tukey’s HSD was applied to ascertain significance. *represents *p* < 0.05 and **represents *p* < 0.01 when comparing OKN-007 to the combination of vehicle addition, OKN-007, and 5-BDBD; + represents *p* < 0.05 and ++ represents *p* < 0.01 when comparing OKN-007 to the combination of vehicle addition, OKN-007, 5-BDBD, and heparan sodium sulfate.

We had previously shown that eATP exerts its cytotoxic effects on TNBC cells through P2RX4 and P2RX7 receptors.^8^ We sought to confirm whether the exaggerated loss of cell viability in the presence of OKN-007 is dependent on eATP-induced activation of P2RX4 or P2RX7 (Figure 8). We chose to examine Hs 578t cells because we observed the largest increase in eATP and loss of cell viability in this cell type when exposed to the combination of paclitaxel, OKN-007, and heparan sodium sulfate. We observed a reversal of the effects of OKN-007 on eATP release and cell viability upon exposure to both the P2RX7 inhibitor A437809 (Figures 8a,b) and the P2RX4 inhibitor 5-BDBD (Figures 8c,d). There was a significant decline in eATP (*p* < 0.0001) and increased cell viability (*p* < 0.0001) when comparing the combination of paclitaxel with OKN-007 and A437809 to that of paclitaxel with OKN-007. In addition, there was a significant decline in eATP (*p* < 0.0001) and increased cell viability (*p* < 0.0001) when comparing the combination of paclitaxel, heparan sodium sulfate, OKN-007, and 5-BDBD to that of paclitaxel, heparan sodium sulfate, and OKN-007. This data reveals that the exaggerated loss of cell viability observed when OKN-007 is combined with paclitaxel is dependent on the activation of P2RX4 and P2RX7 by eATP.

### Purinergic signaling and cancer-initiating cells

**Figure 9.**
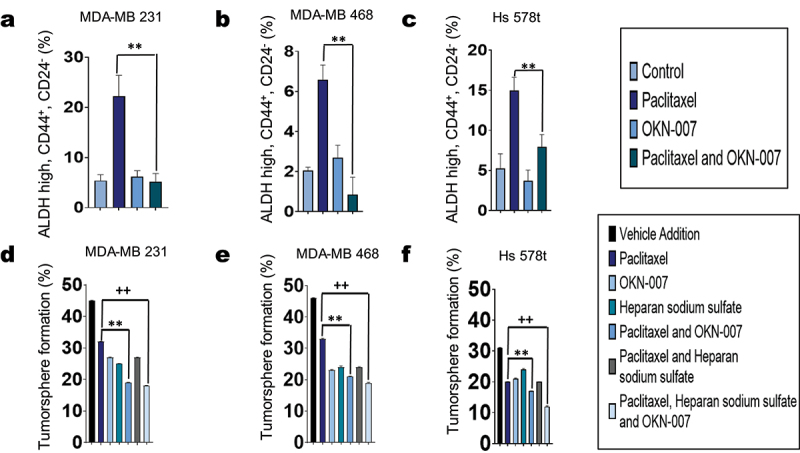
Effect of OKN-007 and chemotherapy on the cancer-initiating cell fraction of TNBC cells. The three TNBC cell lines (a) MDA-MB 231, (b) MDA-MB 468, and (c) Hs 578t were treated with OKN-007 (20 µm, 48 hours) and/or paclitaxel (100 µm, final 6 hours of the 48-hour time course to replicate exposure times in patients). Paclitaxel alone increased the cancer-initiating cell fraction among the surviving cells (cells that express high levels of ALDH and CD44 but do not express CD24), while the combination of OKN-007 and paclitaxel decreased the cancer-initiating cell fraction. One-way ANOVA with Tukey’s HSD was applied to ascertain significance. * represents *p* < 0.05 and **represents *p* < 0.01 when comparing paclitaxel to paclitaxel and OKN-007. Effects on cancer-initiating cells were also determined through the tumorsphere formation efficiency assay in which TNBC cell lines (d) MDA-MB 231, (e) MDA-MB 468, and (f) Hs 578t cells were treated with vehicle (DMSO), paclitaxel (100 µm, final 6 hours of the 48-hour time course to replicate exposure times in patients), heparan sodium sulfate (50 µm, 48 hours), OKN-007 (20 µm, 48 hours), or the different combinations listed. Treated TNBC cells were washed, passed through cell strainers, collected, and plated at approximately one cell per well on round-bottom, low-attachment, 96-well plates; tumorspheres were allowed to form for 7 days. The fraction of wells plated with at least one live cell positive for tumorspheres after 7 days were counted using Etaluma™ Lumascope 620. The combination regimens showed a significant decrease in tumorsphere formation. There was a significant decrease in sphere forming MDA-MB 231, MDA-MB 468 and Hs 578t cells when comparing paclitaxel-treated cells with paclitaxel and OKN-007-treated cells and when comparing paclitaxel-treated cells with paclitaxel, OKN-007 and heparan sulfate-treated cells. One-way ANOVA with Tukey’s HSD was applied to ascertain significance. **represents *p* < 0.01 when comparing paclitaxel to paclitaxel and OKN-007. ++ represents *p* < 0.01 when comparing paclitaxel to paclitaxel, heparan sodium sulfate, and OKN-007.

Additionally, we sought to analyze the impact of the sulfatase inhibitor OKN-007 in combination with the chemotherapeutic agent paclitaxel on cancer-initiating cell properties of treated cells. Breast cancer-initiating cells have previously been shown to express aldehyde dehydrogenase (ALDH) intracellularly and CD44, but not CD24 at the cell surface.^39–42^ Hence, flow cytometry analysis was performed on TNBC cell lines MDA-MB 231, MDA-MB 468, and Hs 578t treated with OKN-007 for 48 hours and paclitaxel (100 µM) for the final 6 hours to determine the fraction of residual cells with a cancer-initiating cell phenotype (cells that express high levels of ALDH and CD44 but do not express CD24). Paclitaxel alone increased the fraction of residual cancer-initiating cells while OKN-007 combined with paclitaxel reduced the fraction of cancer-initiating cells (Figures 9a–c; Supplemental Figures S15-S19).

Furthermore, we applied an orthogonal approach to analyze cancer-initiating cells by conducting tumorsphere formation efficiency assays. TNBC cell lines were treated with paclitaxel, OKN-007, and/or heparan sodium sulfate and then cultured on low-attachment round-bottom plates in sphere-forming medium in the absence of drug. After 7 days, we observed that the fraction of wells with tumorsphere formation decreased in all TNBC cell lines – MDA-MB 231, MDA-MB 468, and Hs 578t – treated with paclitaxel as compared to vehicle control but was lowest when treated with the combination of paclitaxel, OKN-007 and heparan sulfate. Spheroids were visualized using the Etaluma™Lumascope 620 (Figures 9d–f). Images were also recorded (Supplemental Figures S20–S22). This data aligns with flow cytometry analysis in that the sulfatase inhibitor OKN-007 depressed cancer-initiating cell formation. This also further confirms that the sulfatase inhibitor OKN-007 as an effective agent to overcome chemotherapeutic resistance in TNBCs.

## Discussion

Chemotherapy is still the most effective treatment for TNBC. A major drawback of chemotherapy is its failure to eradicate metastatic disease, despite transient responses. Therapeutic strategies that deepen and lengthen responses are urgently needed. eATP, in the high micromolar to millimolar range, is cytotoxic to cancer cell lines. We previously showed that chemotherapy treatment augments eATP release from TNBC cells.^8^ We also showed that ecto-ATPase inhibitors exacerbate chemotherapy-induced eATP release from TNBC cells and accentuate chemotherapy-induced cell death. However, one drawback of attempting to develop therapeutic small-molecule ecto-ATPase inhibitors is the presence of multiple families of ecto-ATPases in humans, each with multiple members.

Polysulfated polysaccharides have been shown to inhibit multiple classes of ecto-ATPases. The endogenous polysulfated polysaccharide HS was shown to attenuate the degradation of eATP. Therefore, we hypothesized that increasing fully sulfated HS in the microenvironment of TNBC cells using SULF2 inhibitors would augment eATP concentrations in the pericellular environment of chemotherapy-treated TNBC cells, and, hence, accentuate chemotherapy-induced cell death.

Furthermore, our immunoblot results show that SULF2 is highly expressed intracellularly and extracellularly in TNBC cell lines in comparison to MCF-10A cells; this was additionally confirmed through ELISAs. Previous publications have suggested that processed SULF2 is primarily an extracellular secreted protein, so we focused on measuring extracellular expression of SULF1 and SULF2.^24,26,37^ Staining results also revealed that there was a difference in the expression of SULF2 at different stages of TNBC, with the most significant difference being between stages 2A and 2B. We did attempt to knock out SULF2 in TNBCs, but the cell viability was diminished, precluding analysis of the effects of SULF2 knock out in TNBC cell lines. This seems to indicate that TNBC cells are dependent on SULF2 for proper proliferation.

As noted previously, SULF1 is selective for 6-O sulfates in the context of trisulfated disaccharide sequences.^30^ It is the 2-O sulfated Iduronic acid—2N, 6-O sulfated glucosamine disaccharide sequence in the “S” domains of heparan sulfate that are selective docking sites for interactions between HS and proteins such as Noggin 1.^30^ We also pointed out that other publications also suggested some degree of difference between SULF1 and SULF2 regarding selectivity for S domains.^13^ Hence, we focused on SULF2 by applying the SULF2 inhibitor OKN-007 because SULF1 has been reported to be a tumor suppressor in certain contexts while SULF2 is exclusively reported to be an oncogene.

Previously, our lab showed that eATP in the high micromolar to millimolar range is toxic to TNBC cells and that inhibitors of each of the major classes of ecto-ATPases exacerbate chemotherapy-induced increases in eATP.^8^ One challenge of this approach as a potential therapeutic strategy is the need to use different inhibitors for each ecto-ATPase class to maximally augment eATP levels. As polysulfated polysaccharides such as HS inhibit all 3 families of ecto-ATPases, we sought to determine the effects of SULF2 inhibitors, which block the desulfation of HS, on chemotherapy-induced augmentation of eATP and chemotherapy-induced TNBC cell death.^8^ After verifying that the SULF2 inhibitor OKN-007 inhibits sulfatase activity at the concentration utilized, we showed that the combination of OKN-007 and chemotherapy (paclitaxel) accentuated extracellular eATP concentrations and enhanced the chemotherapeutic response in TNBC cell lines, leading to a greater loss of viability. Additionally, the effects of the SULF2 inhibitor on eATP levels and TNBC cell death were reversed by specific inhibitors of P2RX4 and P2RX7 eATP receptors, confirming that these effects are dependent on these purinergic receptors; we have previously shown that these receptors are necessary for chemotherapy-induced eATP release from TNBC cells and its cytotoxic effects.^8^

We also examined the effects of combinations of SULF2 inhibitors and chemotherapy on cancer-initiating cells, as failure to eliminate these cells results in the failure of cytotoxic chemotherapy to eradicate metastatic TNBC.^39,41–43^ We found that upon treatment with chemotherapy alone, there was an enrichment of cancer-initiating cells, as assessed by flow cytometry, across all TNBC cell lines, while combination with the sulfatase inhibitor prevented their enrichment in the surviving fraction of cells.

As eATP is a known immune danger signal and its metabolite adenosine a potent immunosuppressant, further work is necessary to determine the immune effects of ecto-ATPase inhibition by HS using immunocompetent in vivo TNBC models. Moreover, further work is necessary to determine if the cytotoxic effects on TNBC cells occur through nonspecific permeability of P2RX7 ion-coupled channels or through downstream activation of pyroptosis by P2RX7. Our future research will focus on this aspect of the effects of HS in the TME. In addition, the precise reasons for the different properties of SULF1 and SULF2, one a tumor suppressor in many biological contexts and the other an established oncogene, need to be determined. The basis for this may be complex and related to the precise identity of the cell surface and extracellular matrix proteins and the location of residues within the HS polymer targeted by each enzyme. Thus, additional work is necessary in this area.

## Conclusion

SULF2 inhibition sensitizes TNBC cell lines to chemotherapy by enhancing eATP concentrations in the microenvironment of chemotherapy-treated cells. Combinations of SULF2 inhibitors with chemotherapy may attenuate the cancer-initiating cell fraction, unlike chemotherapy alone. Thus, SULF2 inhibitors may have the potential to induce deeper and more durable responses when combined with chemotherapy. Moreover, as eATP is a known immune danger signal, it will be critical to evaluate the immune effects of this strategy. Our future goals are to study the effects of SULF2 inhibition in vivo.

## Supplementary Material

Supplemental_Figure_15.JPG

supplemental_figure_legends_Manouchehri.docx

Supplemental_Figure_5.JPG

Supplemental_Figure_18.JPG

Supplemental_Figure_17.JPG

Supplemental_Figure_9.JPG

Supplemental_Figure_20.JPG

Supplemental_Figure_21.JPG

Supplemental_Figure_3.JPG

Supplemental_Figure_12.JPG

Supplemental_Figure_13.JPG

Supplemental_Figure_14.JPG

Supplemental_Figure_4.JPG

Supplemental_Table_1_legend_Manouchehri.docx

Supplemental_Figure_11.JPG

Supplemental_Figure_2.JPG

Supplemental_Figure_19.JPG

Supplemental_Figure_8.JPG

Supplemental_Figure_10.JPG

Supplemental_Figure_16.JPG

Supplemental_Figure_7.JPG

Supplemental_Table_1_no_highlights_Manouchehri.xlsx

Supplemental_Figure_1.JPG

Supplemental_Figure_22.JPG

Supplemental_Figure_6.JPG

## Data Availability

The datasets used and/or analyzed during the current study are available from the corresponding author upon reasonable request.

## Abbreviations

ALDH: aldehyde dehydrogenase
ANOVA: analysis of variance
ATP: adenosine triphosphate
DCIS: ductal carcinoma in situ
DMSO: dimethyl sulfoxide
eATP: extracellular adenosine triphosphate
ELISA: enzyme-linked immunoassay
E-NPPase: ecto-nucleotide pyrophosphatases/phosphodiesterases
ENPP1: ecto-nucleotide pyrophosphatase/phosphodiesterase 1
ENTPD1: ecto-nucleoside triphosphate diphosphohydrolases 1
ER+: estrogen receptor-positive
FACS: fluorescence-activated cell sorting
FITC: fluorescein isothiocyanate
5’-NTs: 5’-nucleotidases
GAPDH: glyceraldehyde-3-phosphate dehydrogenase
HIER: heat-induced epitope retrieval
HER2+: human epidermal growth factor receptor 2-positive
HS: heparan sulfate
HSD: honestly significant difference
MFI: mean fluorescence intensity
mM: millimolar
nM: nanomolar
PAGE: polyacrylamide gel electrophoresis
PBS: phosphate buffered saline
PR+: progesterone receptor-positive
SULF1: sulfatase 1
SULF2: sulfatase 2
TMA: tissue microarray
TME: tumor microenvironment
TNAP: tissue nonspecific alkaline phosphatases
TNBC: triple-negative breast cancer
