# Supplementary figures and images for: Sulfatase 2 inhibition sensitizes triple-negative breast cancer cells to paclitaxel through augmentation of extracellular ATP
Source: Cancer Biol Ther. 2025 Mar 26;26(1):2483989. doi: 10.1080/15384047.2025.2483989 (PMC11951697; doi:10.1080/15384047.2025.2483989)

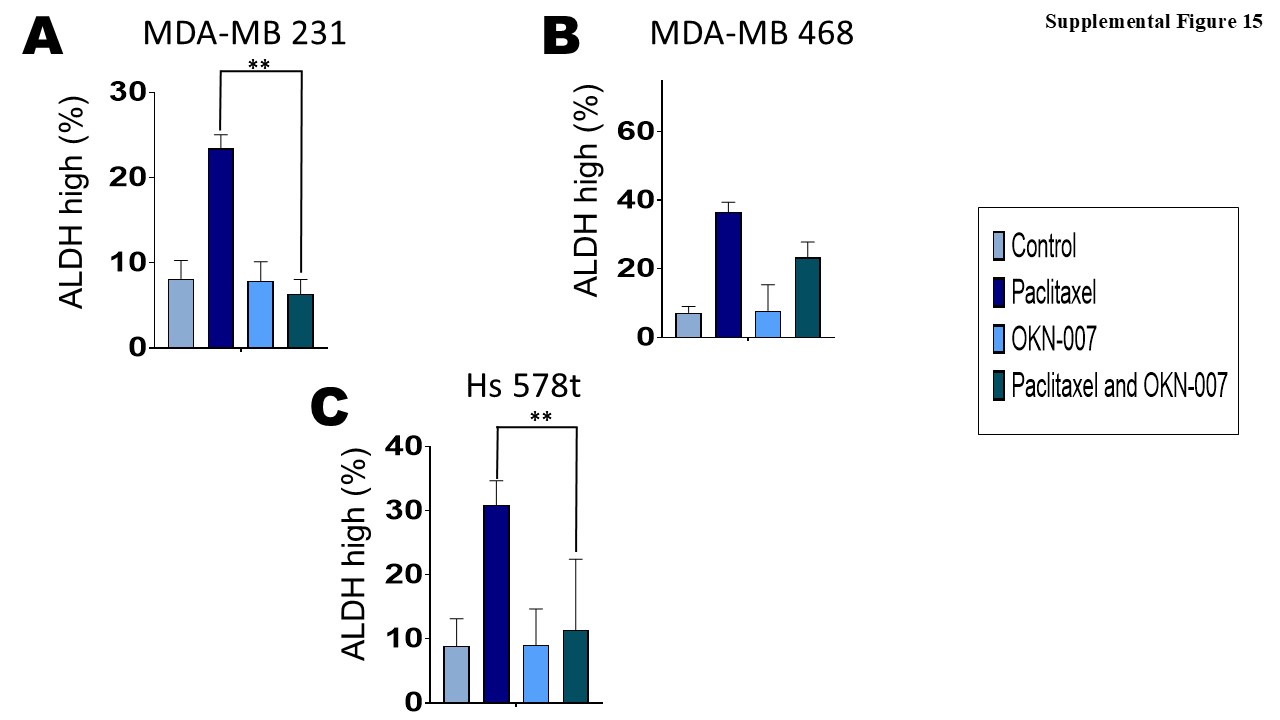

Supplement: Supplemental_Figure_15.JPG [file KCBT_A_2483989_SM5449.jpg]

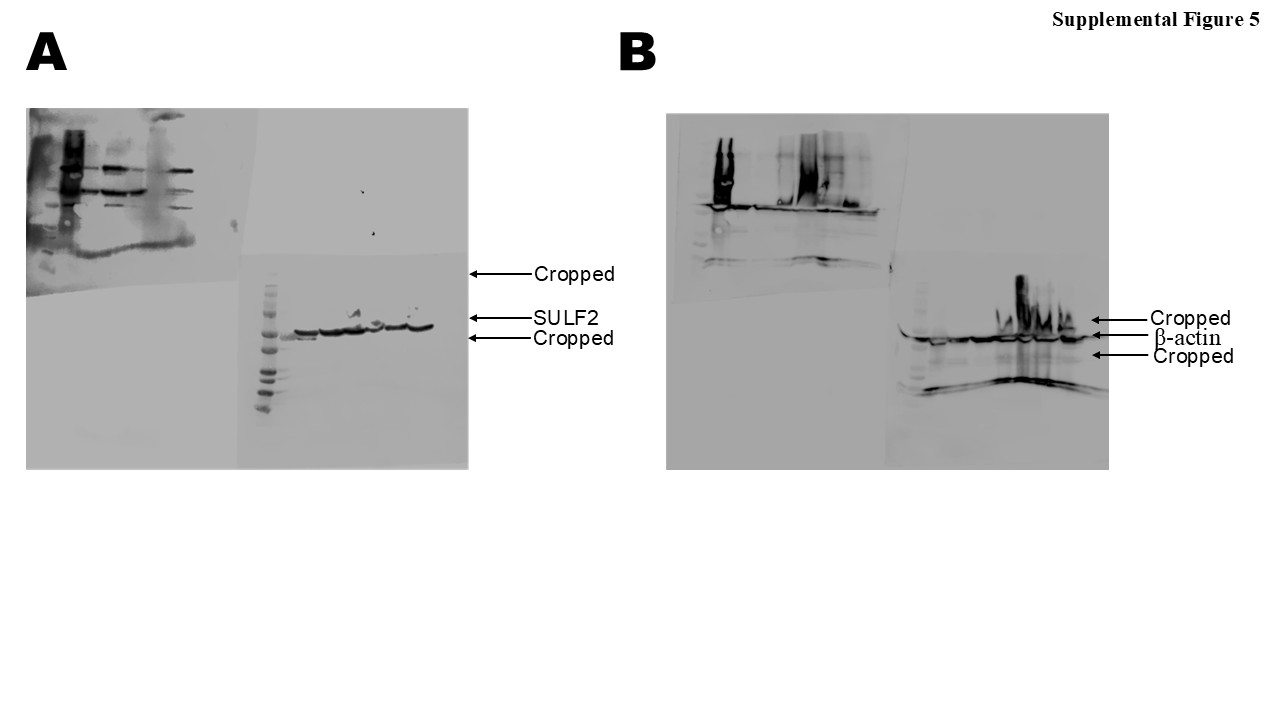

Supplement: Supplemental_Figure_5.JPG [file KCBT_A_2483989_SM5447.jpg]

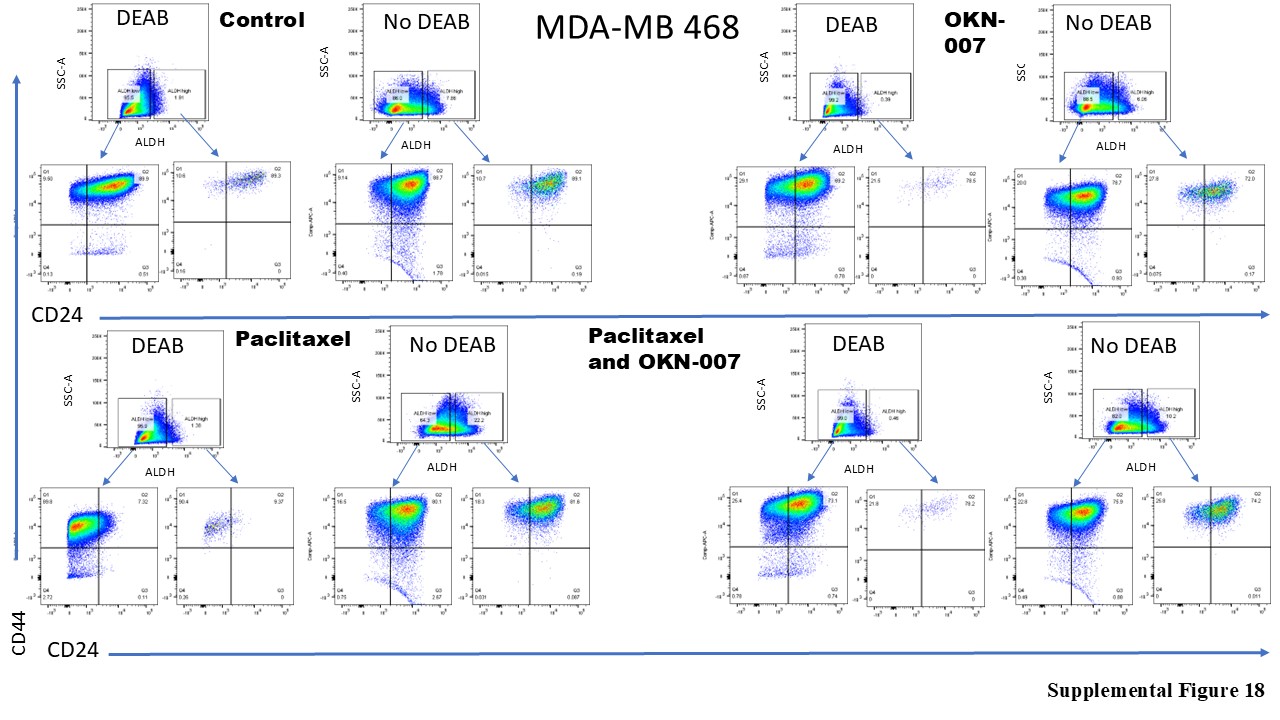

Supplement: Supplemental_Figure_18.JPG [file KCBT_A_2483989_SM5446.jpg]

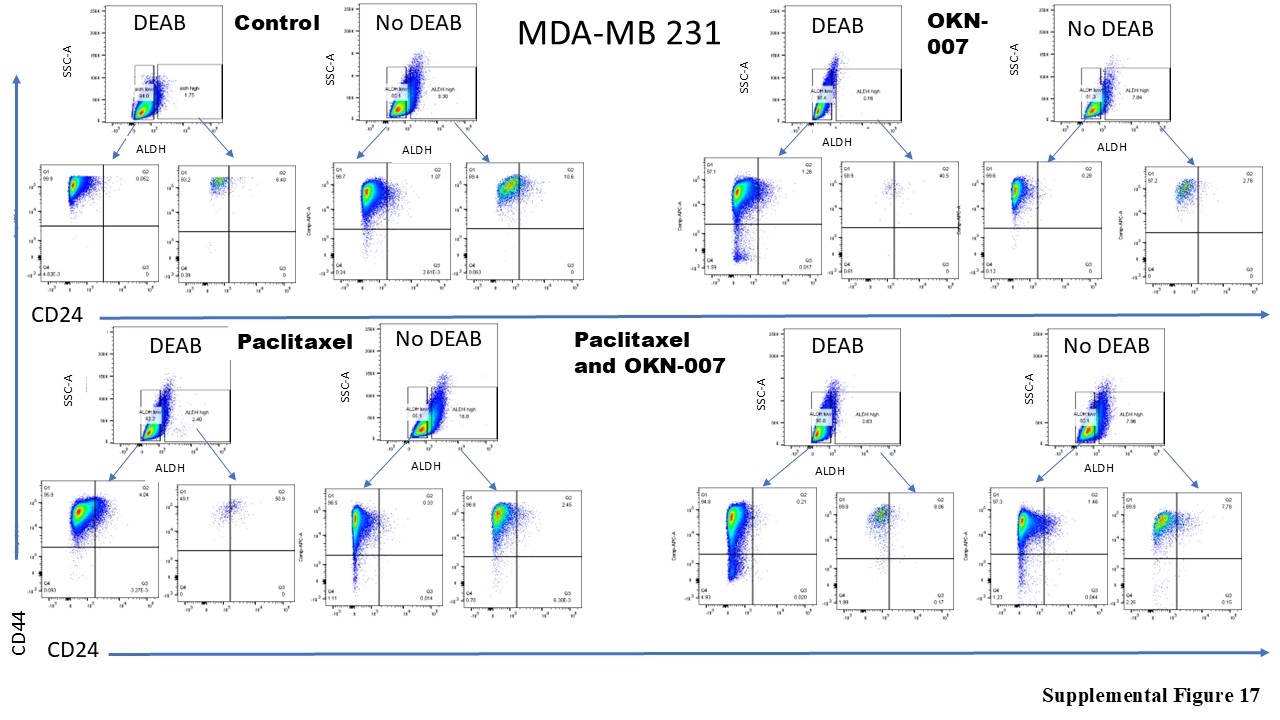

Supplement: Supplemental_Figure_17.JPG [file KCBT_A_2483989_SM5445.jpg]

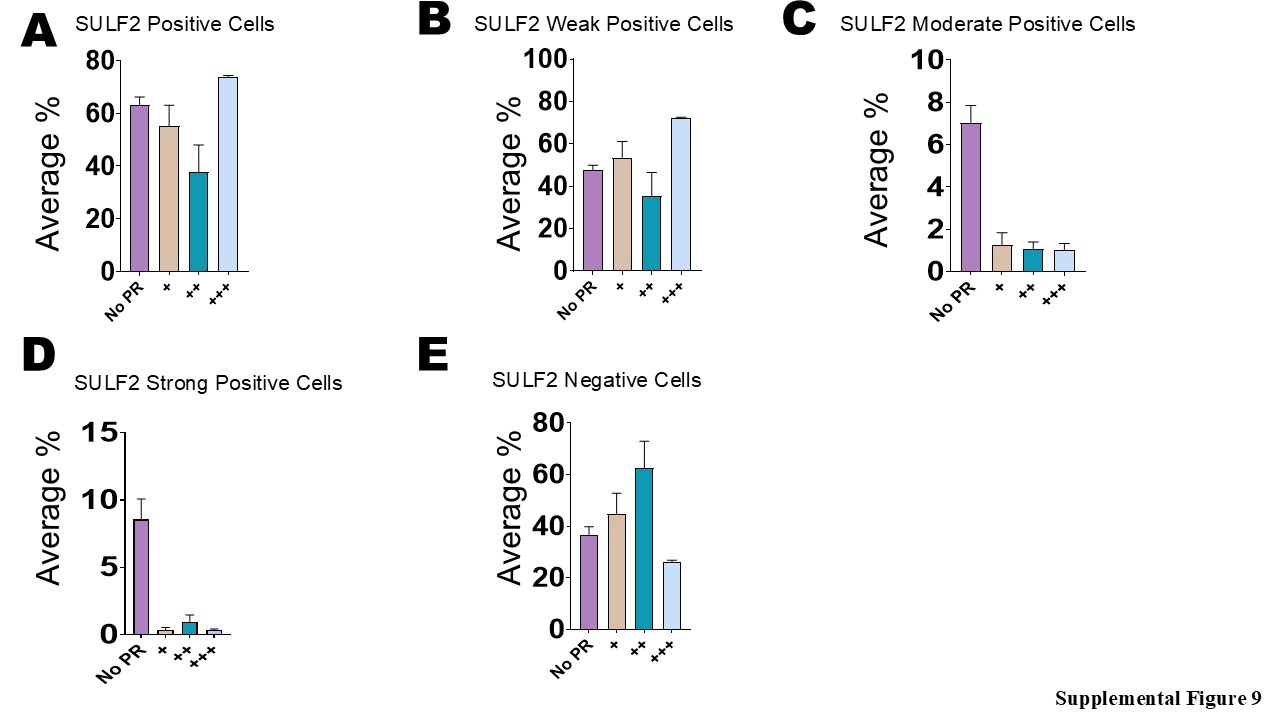

Supplement: Supplemental_Figure_9.JPG [file KCBT_A_2483989_SM5444.jpg]

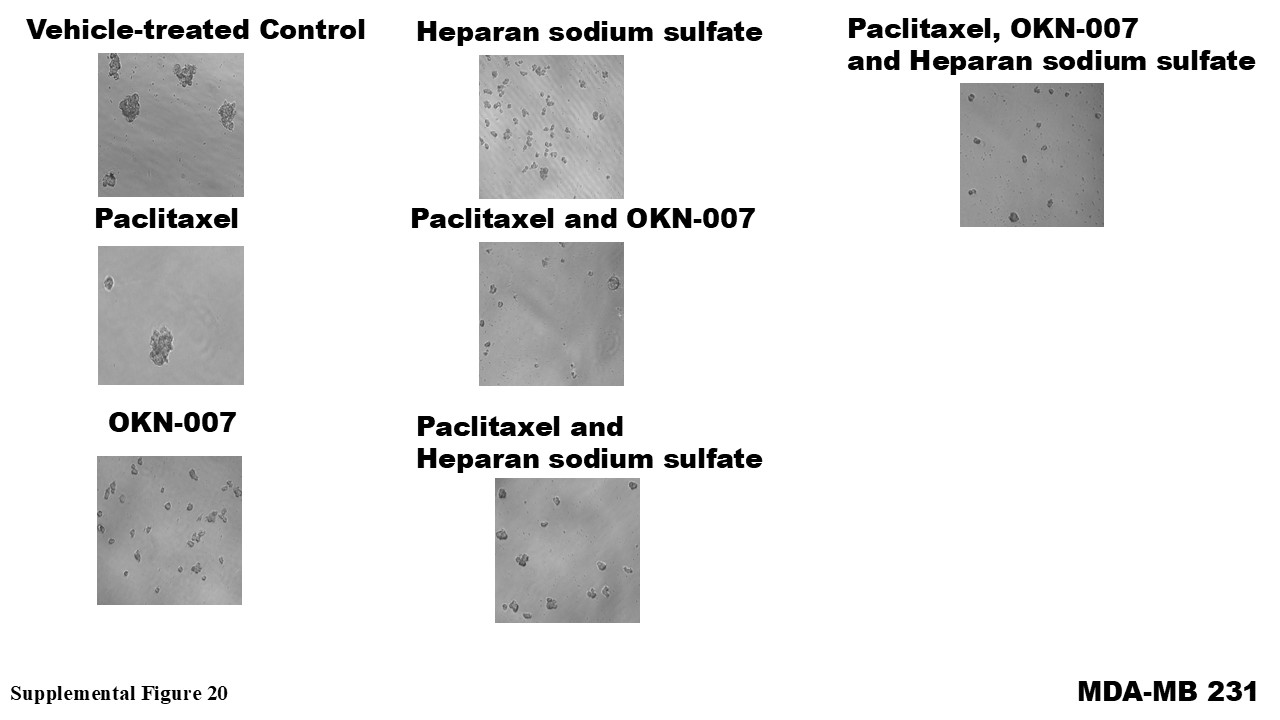

Supplement: Supplemental_Figure_20.JPG [file KCBT_A_2483989_SM5443.jpg]

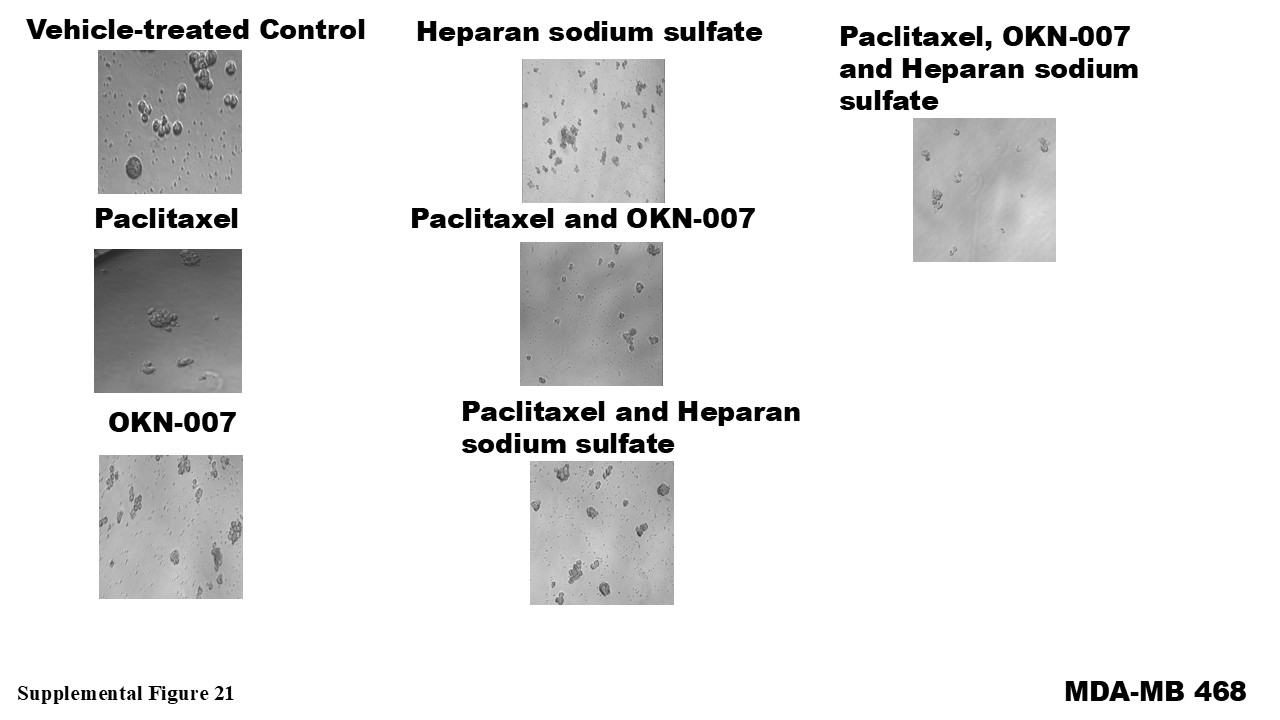

Supplement: Supplemental_Figure_21.JPG [file KCBT_A_2483989_SM5442.jpg]

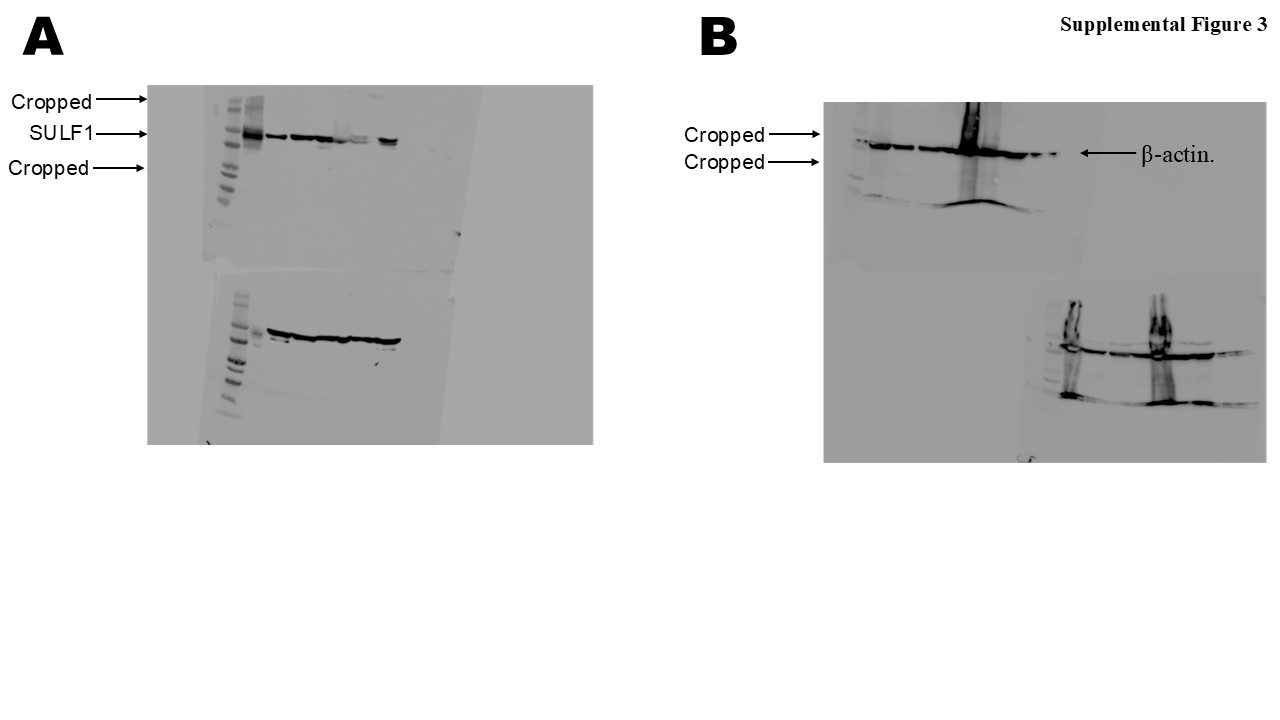

Supplement: Supplemental_Figure_3.JPG [file KCBT_A_2483989_SM5441.jpg]

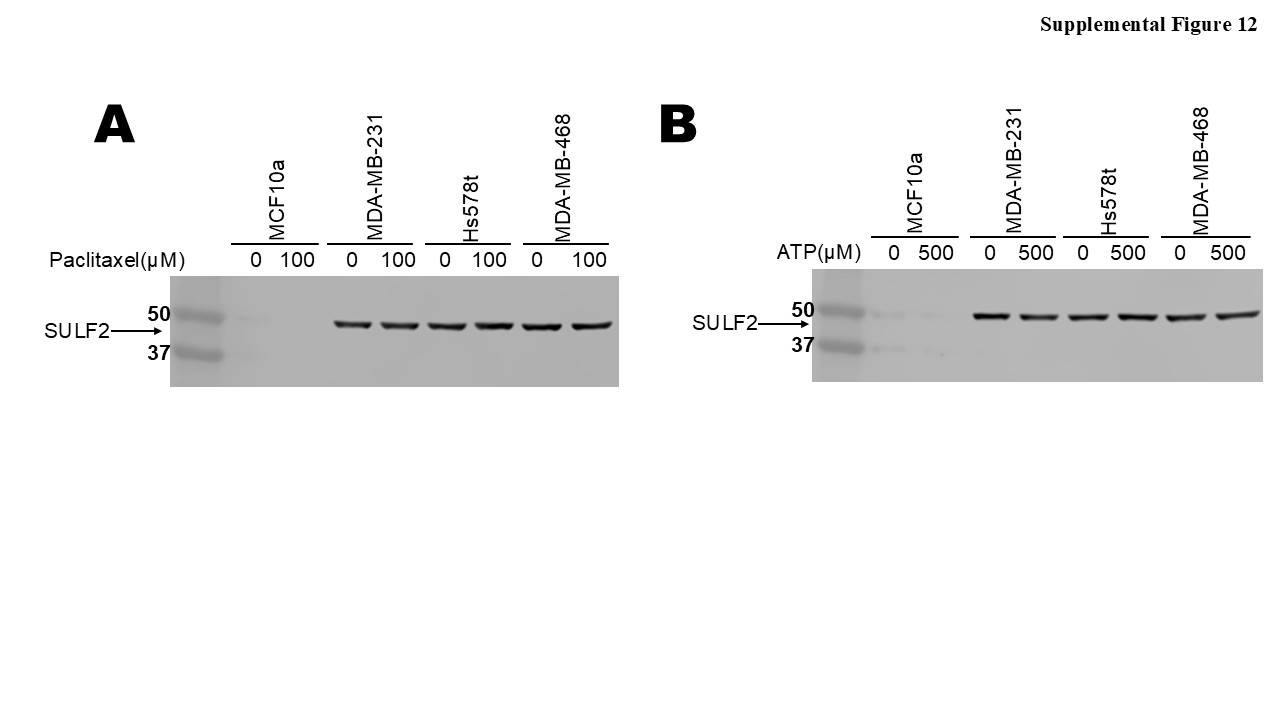

Supplement: Supplemental_Figure_12.JPG [file KCBT_A_2483989_SM5440.jpg]

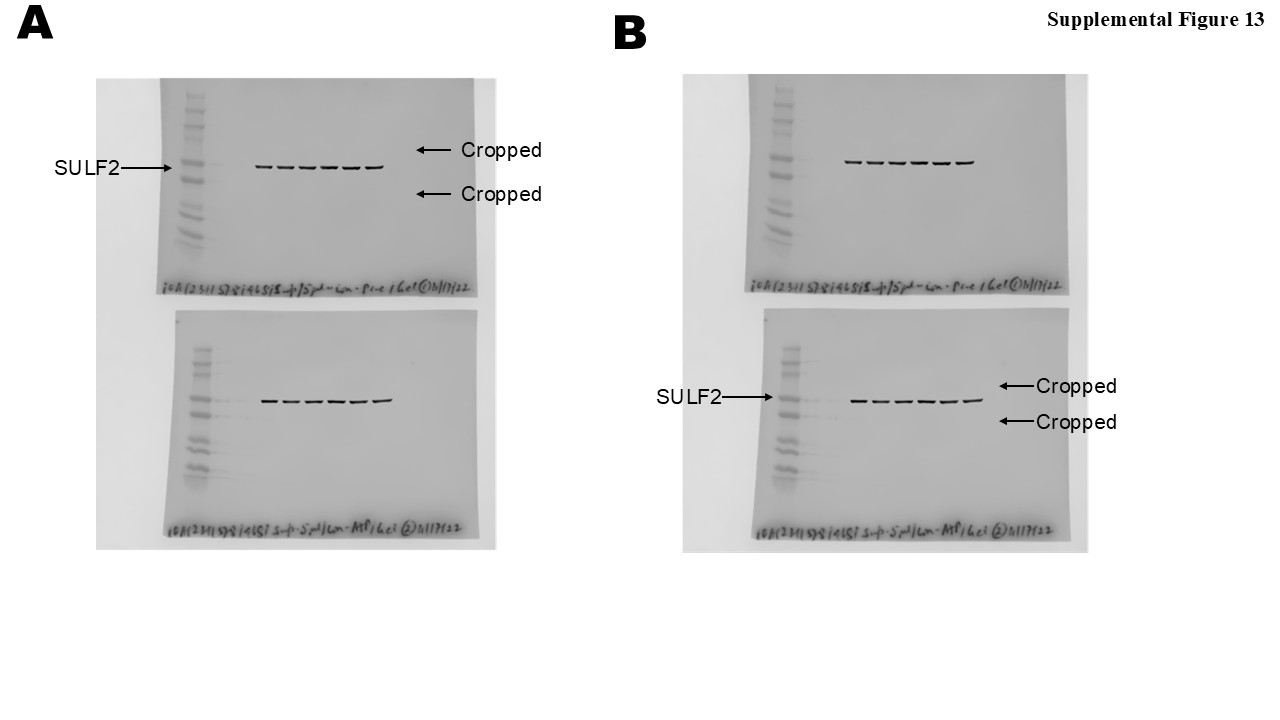

Supplement: Supplemental_Figure_13.JPG [file KCBT_A_2483989_SM5439.jpg]

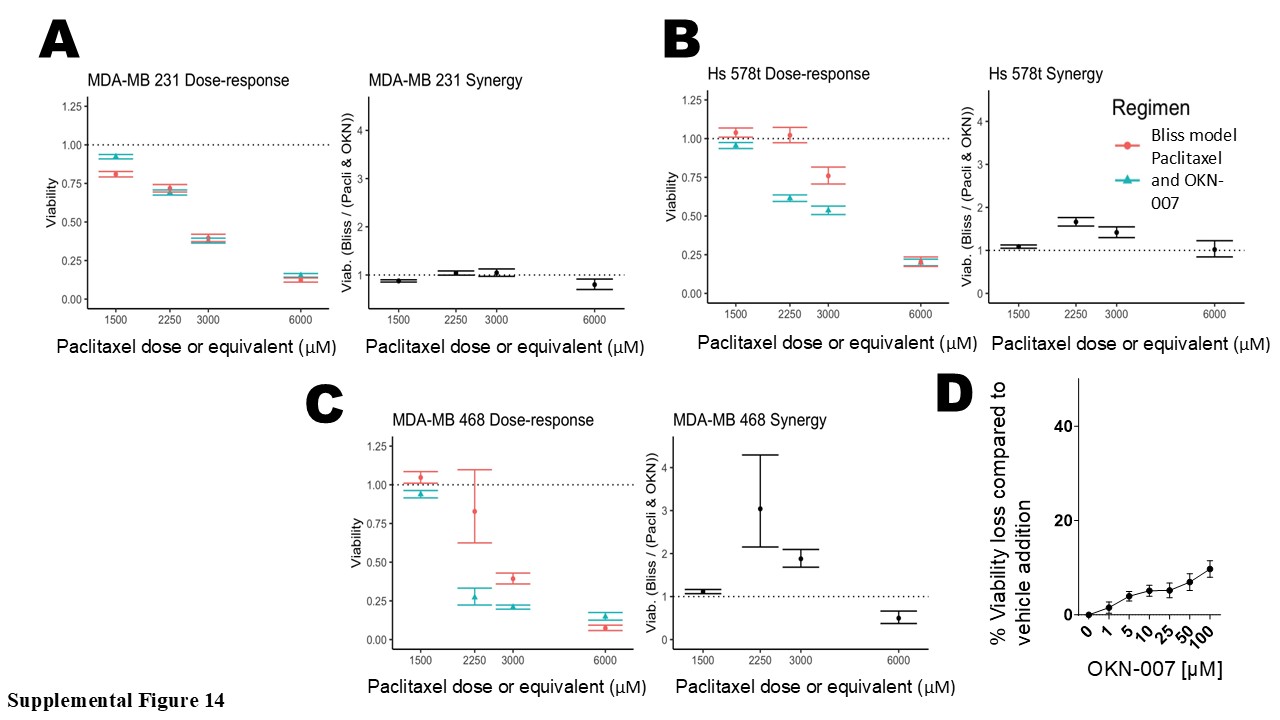

Supplement: Supplemental_Figure_14.JPG [file KCBT_A_2483989_SM5438.jpg]

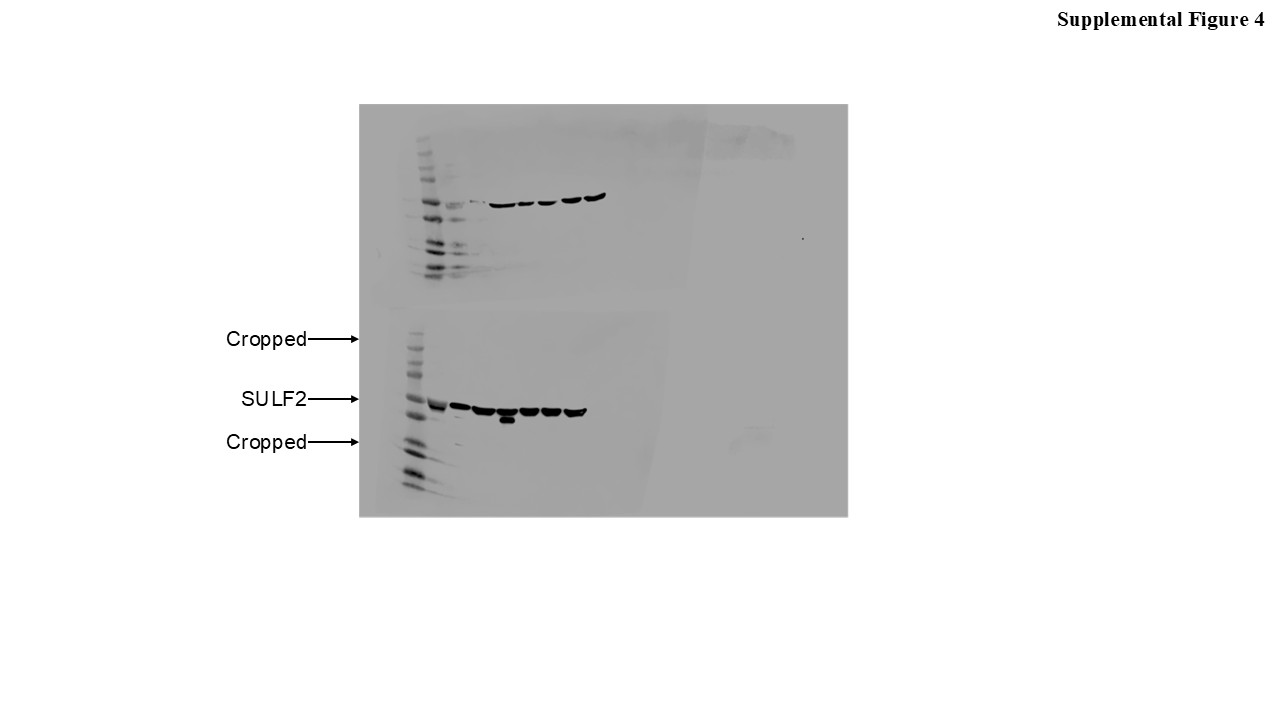

Supplement: Supplemental_Figure_4.JPG [file KCBT_A_2483989_SM5437.jpg]

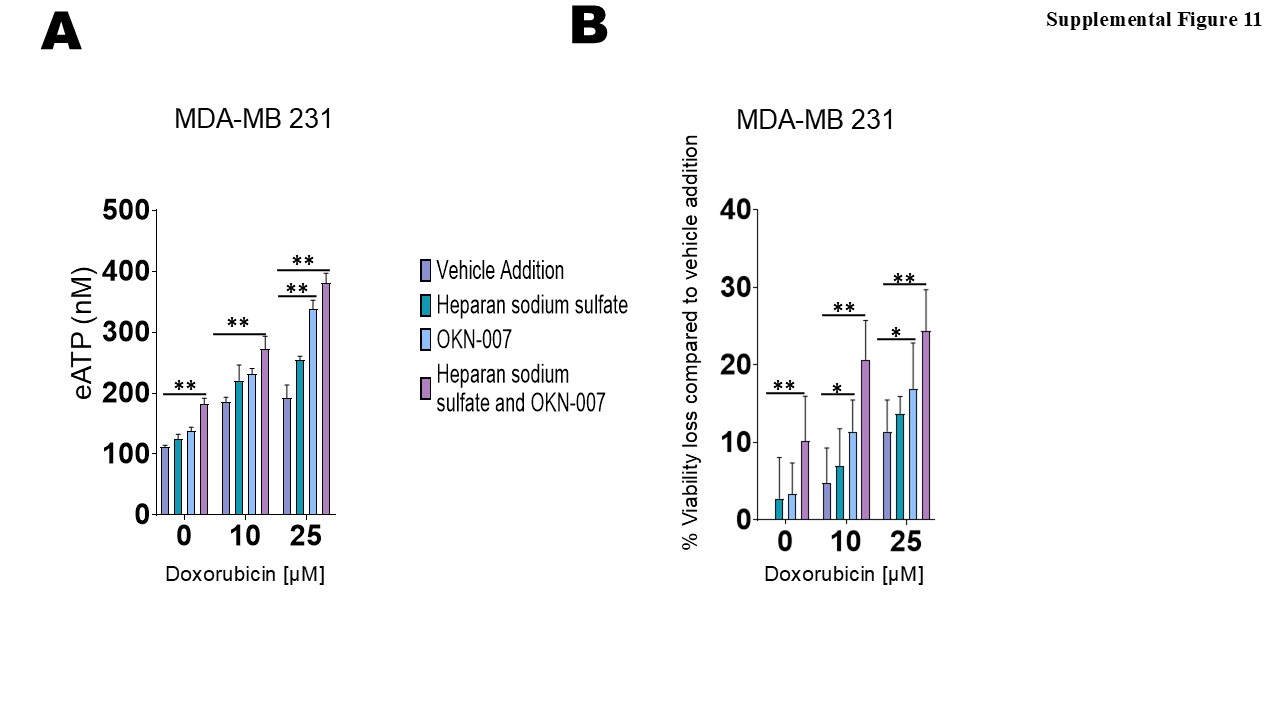

Supplement: Supplemental_Figure_11.JPG [file KCBT_A_2483989_SM5435.jpg]

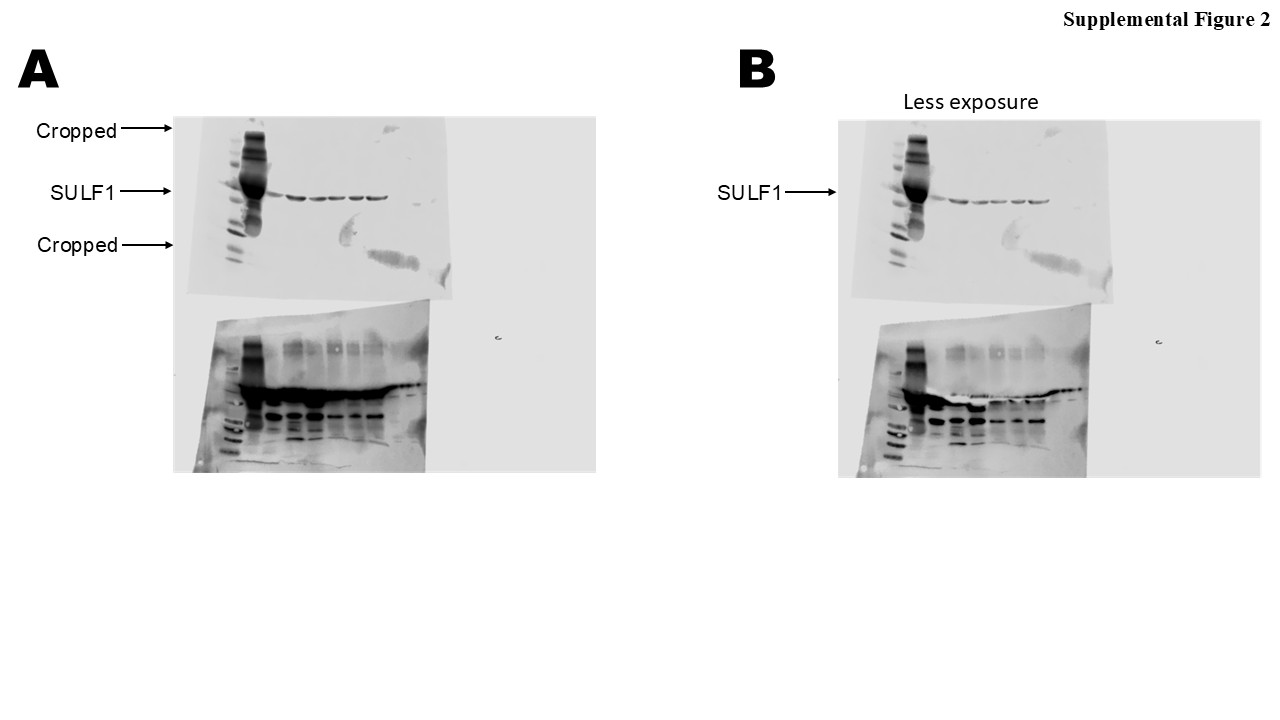

Supplement: Supplemental_Figure_2.JPG [file KCBT_A_2483989_SM5434.jpg]

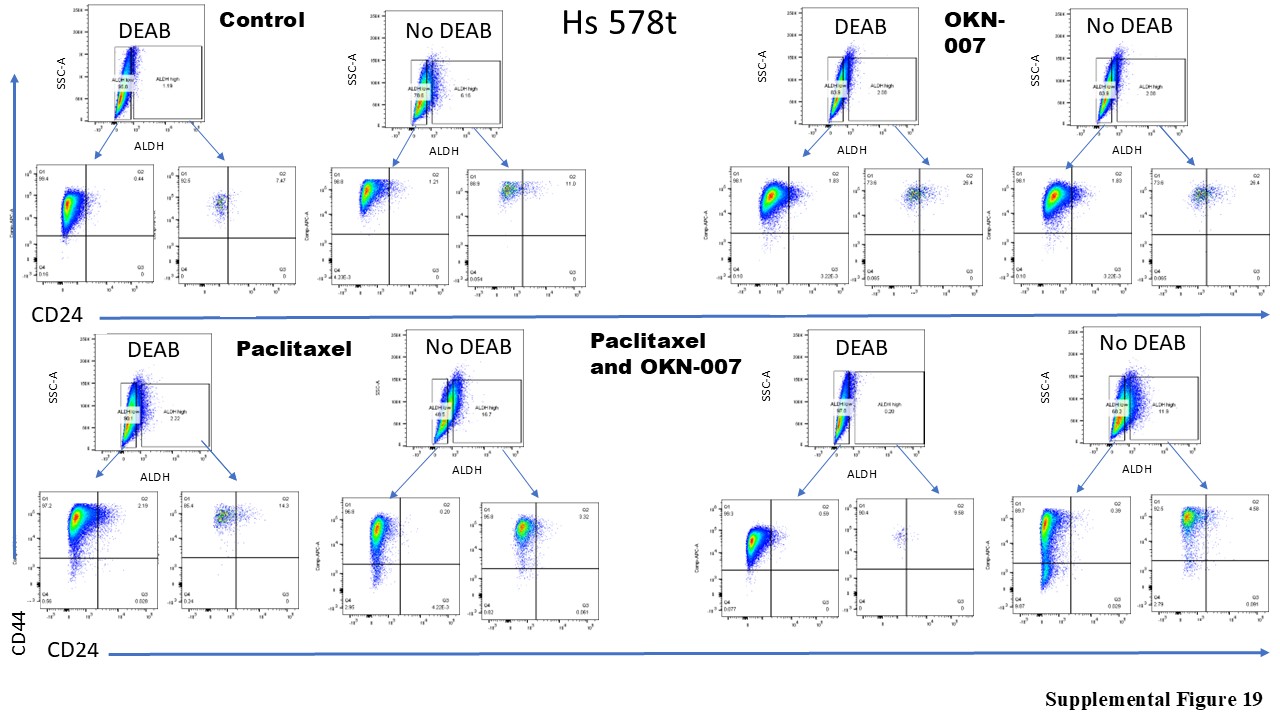

Supplement: Supplemental_Figure_19.JPG [file KCBT_A_2483989_SM5433.jpg]

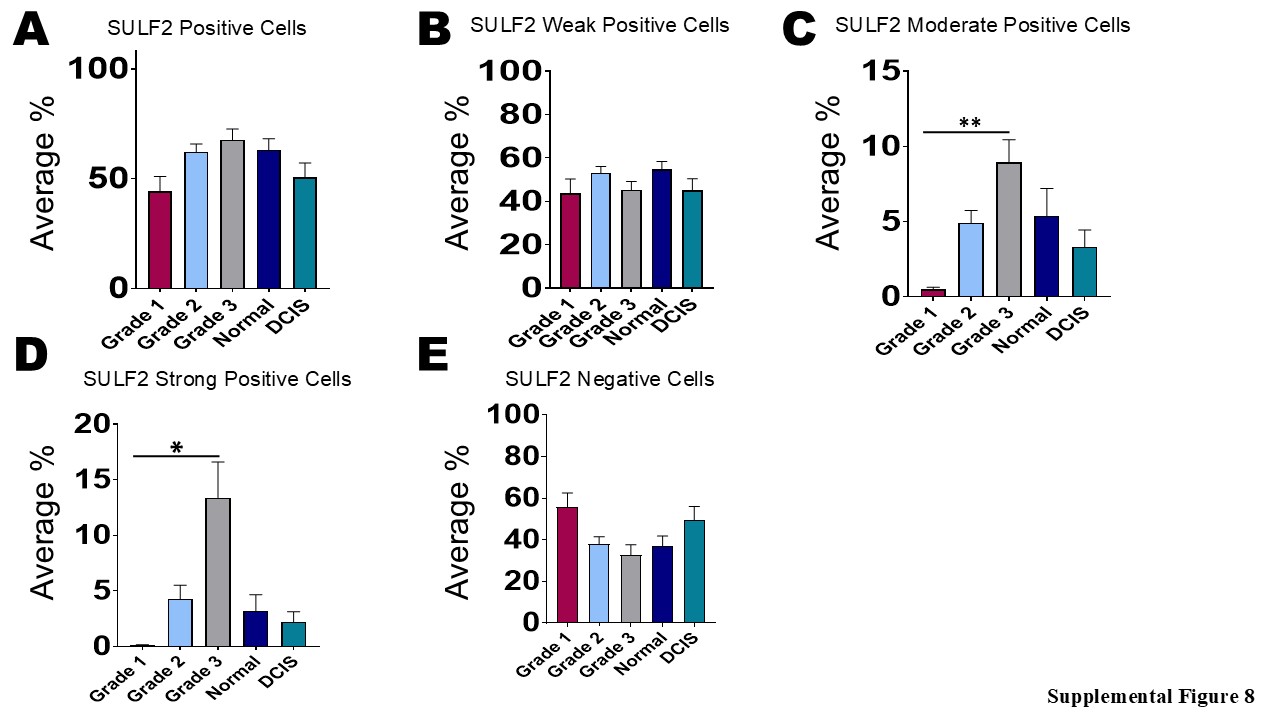

Supplement: Supplemental_Figure_8.JPG [file KCBT_A_2483989_SM5432.jpg]

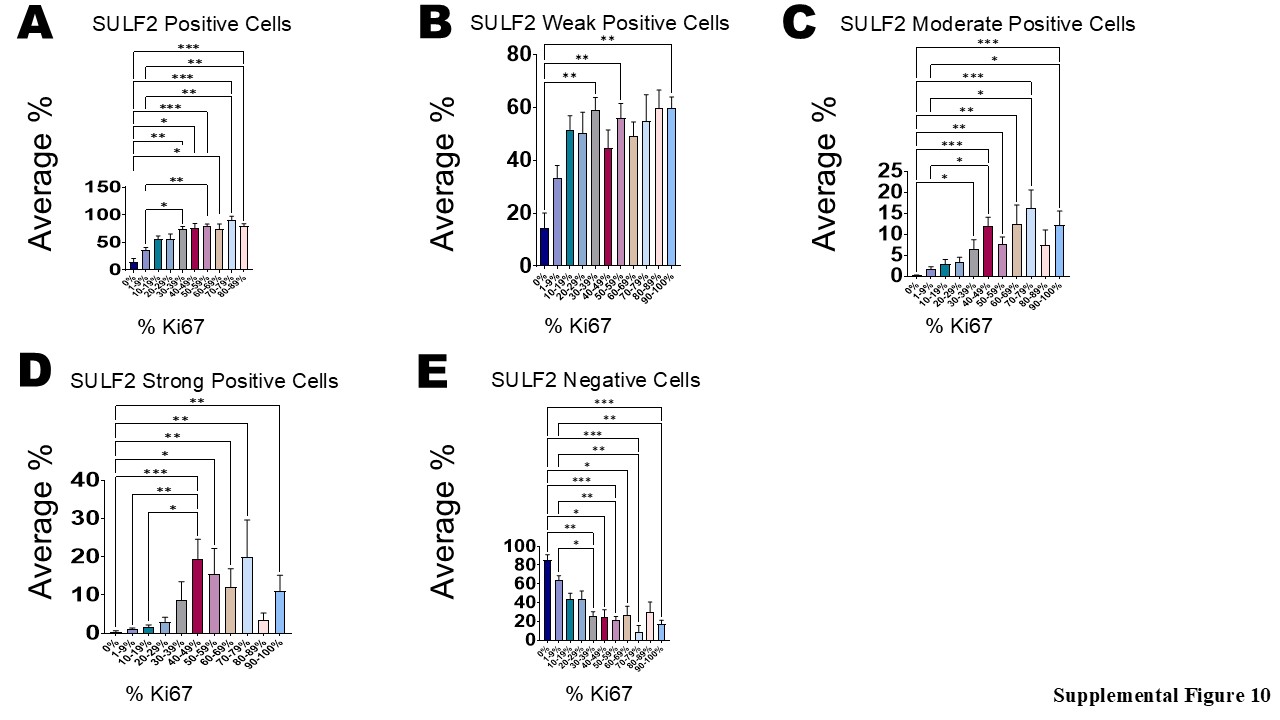

Supplement: Supplemental_Figure_10.JPG [file KCBT_A_2483989_SM5431.jpg]

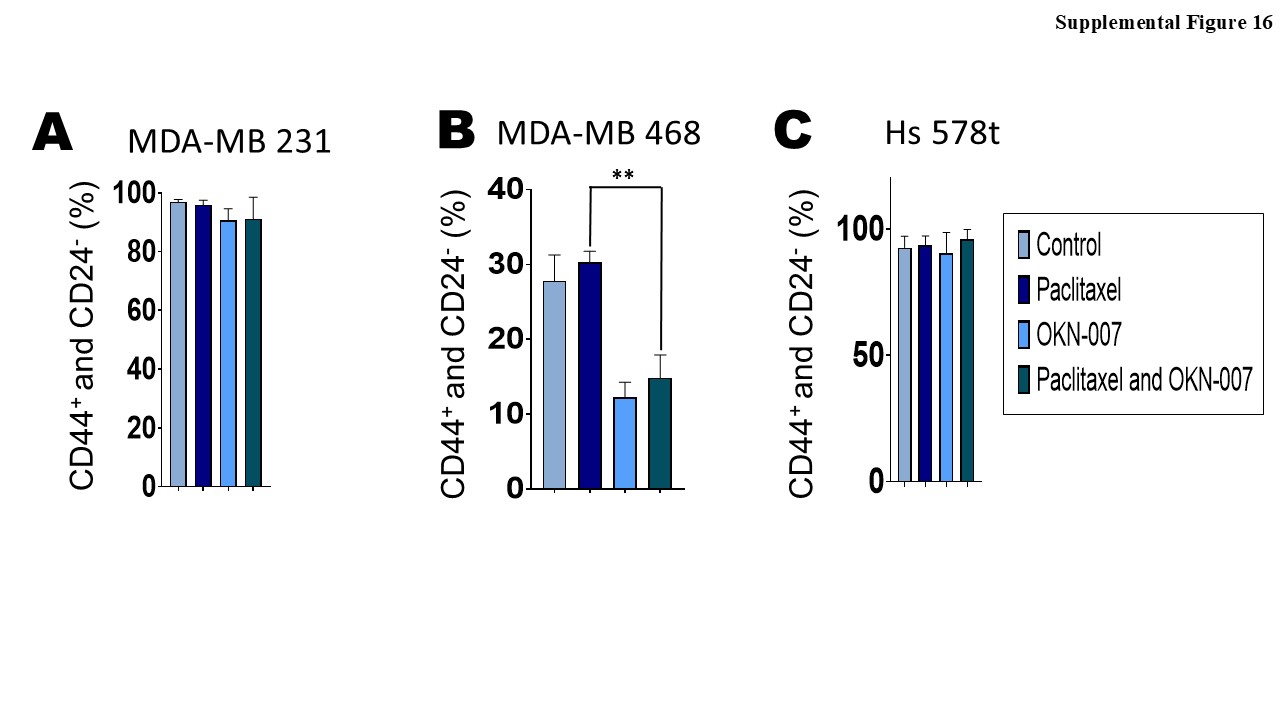

Supplement: Supplemental_Figure_16.JPG [file KCBT_A_2483989_SM5430.jpg]

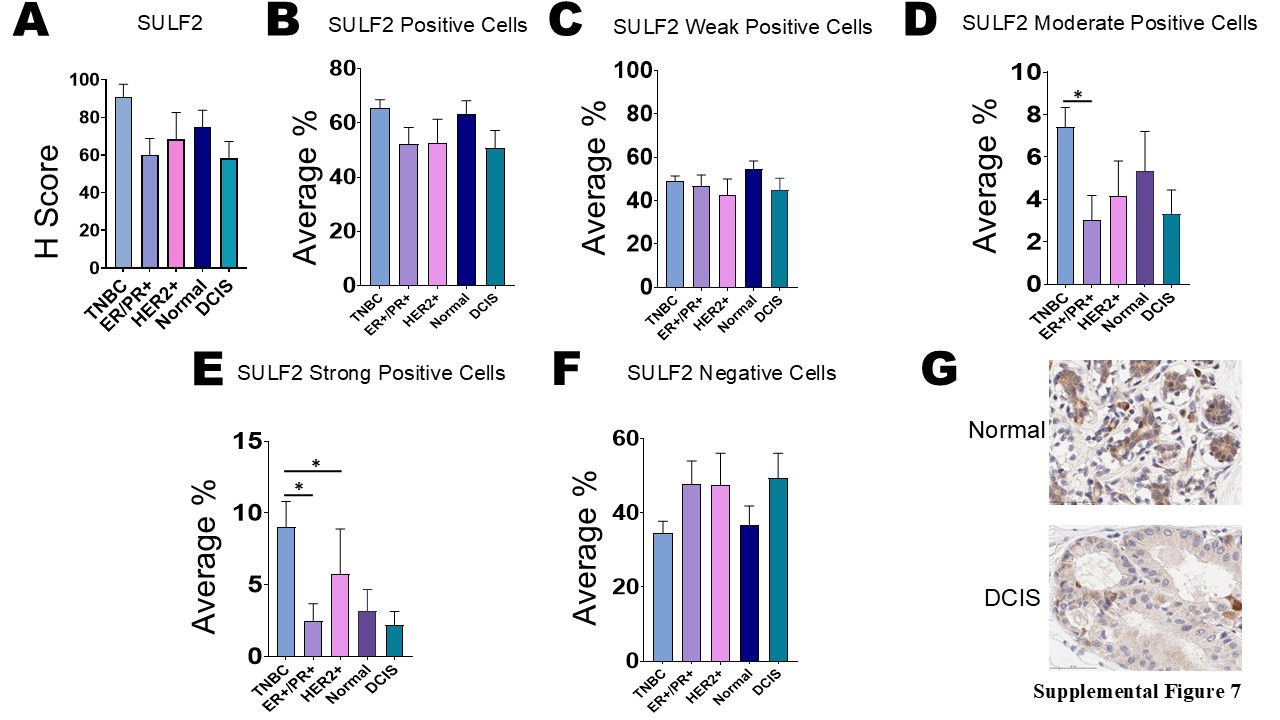

Supplement: Supplemental_Figure_7.JPG [file KCBT_A_2483989_SM5429.jpg]

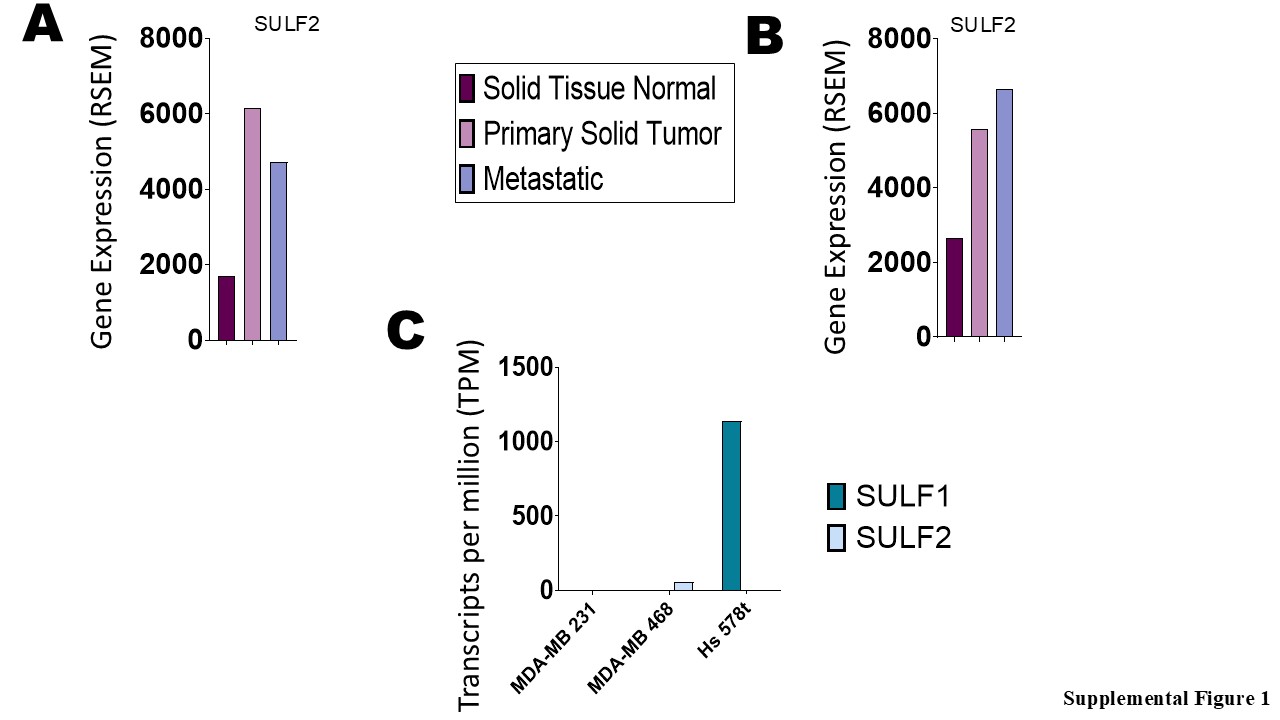

Supplement: Supplemental_Figure_1.JPG [file KCBT_A_2483989_SM5427.jpg]

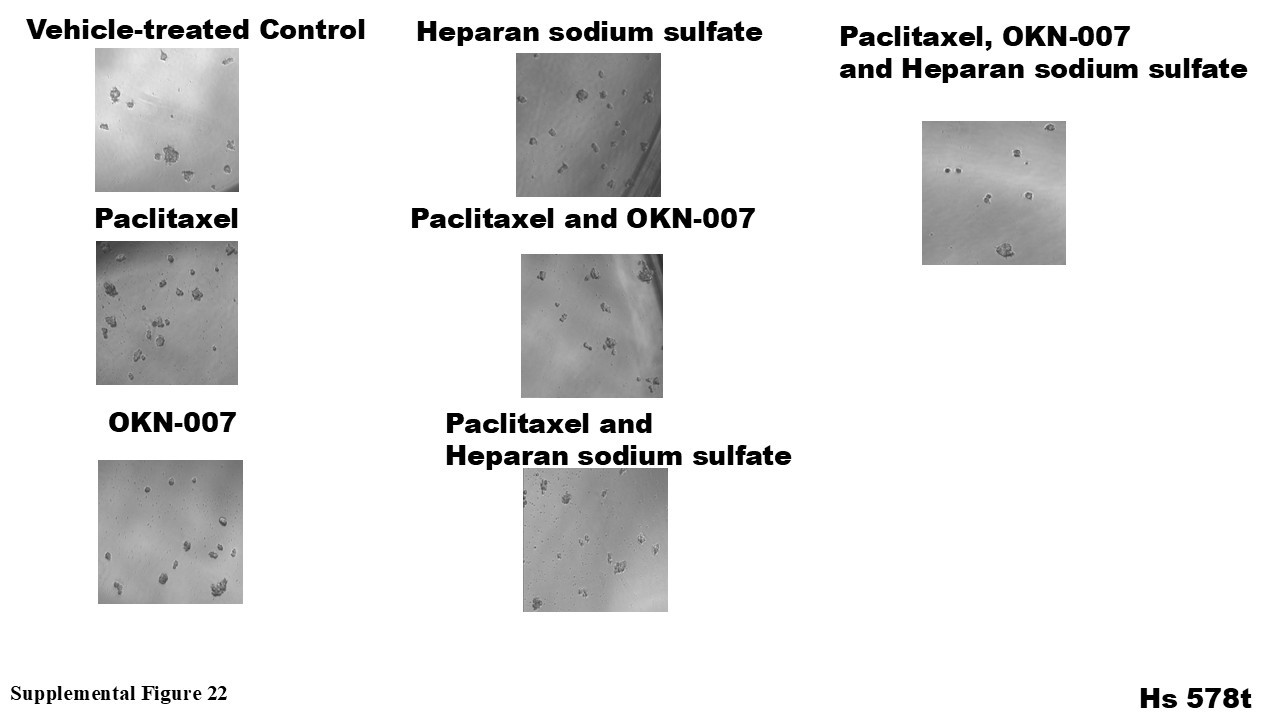

Supplement: Supplemental_Figure_22.JPG [file KCBT_A_2483989_SM5426.jpg]

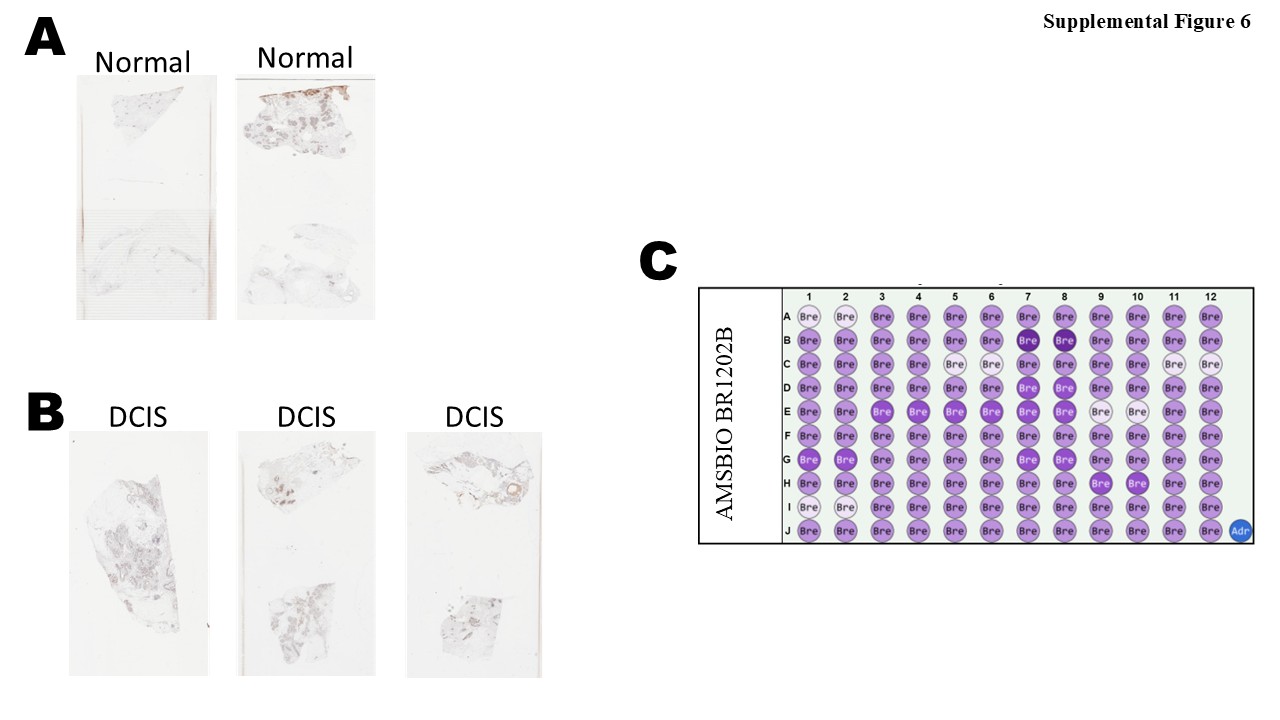

Supplement: Supplemental_Figure_6.JPG [file KCBT_A_2483989_SM5425.jpg]
