# Supplementary material for: Sulfatase 2 inhibition sensitizes triple-negative breast cancer cells to paclitaxel through augmentation of extracellular ATP
Source: Cancer Biol Ther. 2025 Mar 26;26(1):2483989. doi: 10.1080/15384047.2025.2483989 (PMC11951697; doi:10.1080/15384047.2025.2483989)
Supplement: supplemental_figure_legends_Manouchehri.docx [file KCBT_A_2483989_SM5448.docx]

**Supplemental Figure 1: Sulfatase expression analysis.** Expression analysis of **(A)** SULF1 **(B)** SULF2 protein at different stages of breast cancer in RNA-Seq by Expectation-Maximization (RSEM). **(C)** Messenger RNA expression analysis of sulfatases in TNBC MDA-MB 231, Hs 578t and MDA-MB 468 cell lines. This data was obtained from Expression Atlas and TSVdb.

**Supplemental Figure 2:** **Full western blot for SULF1 from Figure 3A. (A)**The full, uncropped western blot for SULF1 from Figure 3A. **(B)** The full, uncropped western blot for a less exposed SULF1 blot from Figure 3A.

**Supplemental Figure 3:** **Full western blot for SULF1** **from Figure 3B.** The full, uncropped western blot for **(A)** SULF1 and **(B)** β-actin from Figure 3B.

**Supplemental Figure 4:** **Full western blot for SULF2 from Figure 3C.** The full, un-cropped western blot for SULF2 from Figure 3C.

**Supplemental Figure 5:** **Full western blot for SULF2** **from Figure 3D.** The full, uncropped western blot for **(A)** SULF2 and **(B)** β-actin from Figure 3D.

**Supplemental Figure 6:** **Immunohistochemistry for SULF2** **staining. (A)** Normal breast tissue on slides (2) stained for SULF2. **(B)** Ductal carcinoma in situ (DCIS) tissue on slides (3) stained for SULF2. **(C)** Slide key for the breast cancer tissue array orientation.

**Supplemental Figure 7.**  **Statistical analysis for SULF2 immunohistochemistry.** **(A)** The Kruskal-Wallis test showed no statistically significant difference in H-score between TNBC, ER+/PR+ breast cancer, HER2+ breast cancer, normal breast tissue, and DCIS. **(B)** The Kruskal-Wallis test indicated that there was no significant difference in the average percentages of cells that stained positively for SULF2 in tissue sections of TNBC, ER+/PR+ breast cancer, HER2+ breast cancer, normal breast tissue, and DCIS. **(C)** The Kruskal-Wallis test results showed that there was no significant difference between the average percentages of cells that stained weakly positive for SULF2 in tissue sections of TNBC, ER+/PR+ breast cancer, HER2+ breast cancer, normal breast tissue, and DCIS. **(D** Pairwise comparisons using Dunn’s test indicated that there was a significant difference between breast cancer sub-types TNBC and ER+/PR+ (p = 0.0199) in the percentages of cells that stained moderately positive for SULF2. No other differences were statistically significantly between breast cancer sub-type TNBC and other groups. **(E)** Pairwise comparisons using Dunn’s test showed that there was a significant difference between the percentages of cells that stained strongly positive for SULF2 between TNBC and ER+/PR+ breast cancer (p = 0.0273) and between TNBC and HER2+ breast cancer (p = 0.0447). There was no statistically significant difference between TNBC and normal breast tissue, and between TNBC and DCIS. **(F)** The Kruskal-Wallis test indicated that there was no significant difference in the percentages of cells that stained negatively for SULF2 among TNBC, ER+/PR+ breast cancer, HER2+ breast cancer, normal breast tissue, and DCIS. Standard error of the mean was calculated. For statistical analysis, * representing p<0.05, ** representing p<0.01 and *** representing p<0.001. **(G)** Images were taken of SULF2-stained normal and DCIS slides on an Evos FL Auto 2 microscope (40×).

**Supplemental Figure 8: Statistical analysis for SULF2** **amongst normal tissue, DCIS, and various grades of cancer. (A)** Pairwise comparisons using Dunn’s test showed that there was no significant difference between the average percentages of cells showing any level of staining for SULF2 in the tissue sections of normal tissue, DCIS, and various grades of cancer**. (B)** Kruskal-Wallis test demonstrated that there was no significant difference between the average percentages of cells showing any level of staining for SULF2 in the tissue sections of normal tissue, DCIS, and various grades of cancer. **(C)** Pairwise comparisons using Dunn’s test indicated that there was a significant difference between percentages of cells staining moderately for SULF2 in tissue sections of cancer grades 1 and 3 (p = 0.01) with grade 3 expressing more SULF2. No other differences were statistically significant. **(D)** Pairwise comparisons using Dunn’s test indicated that there was a significant difference between the percentages of cells staining strongly for SULF2 in tissue sections of cancer grades 1 and 3 (p = 0.0207) with grade 3 expressing more SULF2 than grade 1. **(E)** Pairwise comparisons using Dunn’s test indicated that there was no significant difference between the percentages of cells in the tissue sections of normal tissue, DCIS, and various grades of cancer that were negative for staining for SULF2 between. Standard error of the mean was calculated. For statistical analysis, * representing p<0.05, ** representing p<0.01 and *** representing p<0.001.

**Supplemental Figure 9:** **Additional statistical analysis for SULF2** **staining amongst different PR expression levels. (A)** Kruskal-Wallis test demonstrated that there was no significant difference between the average percentages of cells with any level of staining for SULF2 in tissue sections of breast cancers with different PR expression levels. **(B)** The Kruskal-Wallis test demonstrated that there was no significant difference between the average percentages of cells in tissue sections staining weakly for SULF2 amongst breast cancers expressing different PR levels. **(C)** Pairwise comparisons using Dunn’s test showed that there was no significant difference between the average percentages of cells with moderate staining for SULF2 amongst breast cancers with different PR expression levels. **(D)** Pairwise comparisons using Dunn’s test demonstrated that there was no significant difference between the percentages of cells staining strongly for SULF2 in tissue sections of breast cancers that expressed different PR levels. **(E)** Kruskal-Wallis test demonstrated that there was no significant difference between the percentages of cells that stained negatively for SULF2 in tissue section of breast cancers with different PR expression levels. Standard error of the mean was calculated. For statistical analysis, * representing p<0.05, ** representing p<0.01 and *** representing p<0.001.

**Supplemental Figure 10: Additional statistical analysis for SULF2** **staining and comparing % Ki67 expression levels. (A)** Pairwise comparisons using Dunn’s test indicated that there was a significant difference between the percentages of cells expressing any level of SULF2 in tissue sections of cancers with different levels of Ki67 expression. The higher % Ki67 expressed more SULF2. **(B)** Pairwise comparisons using Dunn’s test indicated that there was a significant difference between the percentages of cells with weak positive staining for SULF2 in tissue sections of cancers with differing Ki67 expression levels. The higher % Ki67 expressed more SULF2. **(C)** Pairwise comparisons using Dunn’s test indicated that there was a significant difference between the percentage of cells with moderately positive staining for SULF2 in tissue sections of cancers with differing Ki67 expression levels. The higher % Ki67 expressed more SULF2. **(D)** Pairwise comparisons using Dunn’s test indicated that there was a significant difference between the percentages of cells staining strongly positive for SULF2 in tissue sections of cancers with differing Ki67 expression levels. The higher % Ki67 expressed more SULF2 **(E)** Pairwise comparisons using Dunn’s test indicated that there was a significant difference between the percentages of cells that were negative for SULF2 staining in tissue sections of cancers with differing Ki67 expression levels. Standard error of the mean was calculated. For statistical analysis, * representing *p*<0.05, ** representing *p*<0.01 and ***p<0.001.

**Supplemental Figure 11: Effects of sulfatase inhibitor OKN-007 combined with chemotherapeutic agent doxorubicin on eATP and cell viability. (A)** eATP and **(B)** % loss of cell viability was measured in treated MDA-MB 231 cells. The treatments applied were vehicle addition (doxorubicin, light purple), heparan sodium sulfate (50 µM, teal), and OKN-007 (20 µM, light blue) or the combination (pink-purple); heparan sodium sulfate and OKN-007 were administered for 48 hours, and doxorubicin was added for the final 6 hours to replicate doxorubicin exposure times in patients. Three independent experiments were performed in triplicate. One-way ANOVA with Tukey’s HSD was applied to ascertain significance. * represents *p*<0.05 and ** represents *p*<0.01 when comparing vehicle addition to heparan sodium sulfate, OKN-007, or the combination.

**Supplemental Figure 12: Western blot analysis for TNBC and MCF-10A cells treated with paclitaxel or ATP**. TNBC MDA-MB 231, Hs 578t and MDA-MB 468 cells and nontumorigenic immortal mammary epithelial MCF-10A cells were treated with **(A)** paclitaxel (100 µM) for 6 hours or **(B)** ATP (500 µM) for 48 hours and 5 μl cell supernatants were probed SULF2. Similar results were obtained in biological replicate experiments.

**Supplemental Figure 13: Full western blot for SULF2 from Supplemental Figure 12A. (A)** The full, un-cropped western blot for SULF2 from Supplemental Figure 12A. **(B)** The full, un-cropped western blot for SULF2 from Supplemental Figure 12B.

**Supplemental Figure 14: Statistical analysis of treated cells with sulfatase inhibitor OKN-007 and chemotherapeutic agent paclitaxel.** Dose response and synergy graphs are displayed for TNBC **(A)** MDA-MB 231 **(B)** Hs 578t and **(C)** MDA-MB 468 with increasing concentrations of paclitaxel, OKN-007 or the co-treatment administered for 48 hours. For the dose response graphs, the bliss model (orange) and the co-treatment (paclitaxel and OKN-007) are shown. There is some synergy (<0.1-1.0) for some dose combinations for MDA-MB 231 cells while there were some drug dose combinations that were additive (1-1.2) for Hs 578t and MDA-MB 468 cells. These graphs were obtained from three independent experiments performed in triplicate. **(D)** Dose-response curve for increasing concentrations of OKN-007.

**Supplemental Figure 15: ALDH expression in TNBC cells.** The percent of cells is shown for **(A)** MDA-MB 231 **(B)** MDA-MB 468 and **(C)** Hs 578t that are considered ALDH high. TNBCs treated with paclitaxel alone produced the most cells that expressed ALDH at high concentrations. Standard deviation was calculated from three independent experiments performed in triplicate. One-way ANOVA with Tukey’s HSD was applied to ascertain significance. * represents p<0.05 and ** represents p<0.01 when comparing paclitaxel to paclitaxel and OKN-007.

**Supplemental Figure 16:** **CD44 and CD24 expressions in TNBC cells.** The percent of cells is shown for **(A)** MDA-MB 231 **(B)** MDA-MB 468 and **(C)** Hs 578t that are considered CD44 positive and CD24 negative. MDA-MB 231 and Hs 578t cells highly express CD44 under all drug conditions; whereas, MDA-MB 468 cells treated with vehicle or paclitaxel expressed more CD44 than those treated with OKN-007 or the co-treatment of OKN-007 and paclitaxel. Standard deviation was calculated from three independent experiments performed in triplicate. One-way ANOVA with Tukey’s HSD was applied to ascertain significance. * represents p<0.05 and ** represents p<0.01 when comparing paclitaxel to paclitaxel and OKN-007.

**Supplemental Figure 17:** **Cancer-initiating cell dot plots for OKN-007 and paclitaxel-treated MDA-MB 231 cells**. Dot plots examining the cancer-initiating cells that are ALDH high, CD44 positive, and CD24 negative are presented for treated MDA-MB 231 cells with paclitaxel, sulfatase inhibitor OKN-007, or both drug agents. Diethylaminobenzaldehyde (DEAB) is a specific inhibitor of ALDH, that can be used as a background fluorescence control. Three independent experiments were performed in triplicate.

**Supplemental Figure 18:** **Cancer-initiating cell dot plots for OKN-007 and paclitaxel-treated MDA-MB 468**. Dot plots examining the cancer-initiating cells that are ALDH high, CD44 positive, and CD24 negative are presented for treated MDA-MB 468 cells with paclitaxel, sulfatase inhibitor OKN-007, or both drug agents. DEAB is used as a background fluorescence control. Three independent experiments were performed in triplicate.

**Supplemental Figure 19:** **Cancer-initiating cell dot plots for OKN-007 and paclitaxel-treated Hs 578t cells**. Dot plots examining the cancer-initiating cells that are ALDH high, CD44 positive, and CD24 negative are presented for treated Hs 578t cells with paclitaxel, sulfatase inhibitor OKN-007, or both drug agents. DEAB is used as a background fluorescence control. Three independent experiments were performed in triplicate.

**Supplemental Figure 20: Tumorsphere efficiency assay images for treated MDA-MB 231 cells**. Tumorsphere images obtained from the Etaluma™ Lumascope 620 (10X) are displayed for each treatment of MDA-MB 231 cells with paclitaxel, OKN-007, heparan sodium sulfate, or the different combinations. Three independent experiments were performed in triplicate.

**Supplemental Figure 21: Tumorsphere efficiency assay images for treated MDA-MB 468 cells**. Tumorsphere images obtained from the Etaluma™ Lumascope 620 (10X) are displayed for each treatment of MDA-MB 468 cells with paclitaxel, OKN-007, heparan sodium sulfate, or the different combinations. Three independent experiments were performed in triplicate.

**Supplemental Figure 22:** **Tumorsphere efficiency assay images for treated Hs 578t cells**. Tumorsphere images obtained from the Etaluma™ Lumascope 620 (10X) are displayed for each treatment of Hs 578t cells with paclitaxel, OKN-007, heparan sodium sulfate, or the different combinations. Three independent experiments were performed in triplicate.
