# Supplementary material for: Sulfatase 2 inhibition sensitizes triple-negative breast cancer cells to paclitaxel through augmentation of extracellular ATP
Source: Cancer Biol Ther. 2025 Mar 26;26(1):2483989. doi: 10.1080/15384047.2025.2483989 (PMC11951697; doi:10.1080/15384047.2025.2483989)
Supplement: Supplemental_Table_1_legend_Manouchehri.docx [file KCBT_A_2483989_SM5436.docx]

**Supplemental Table 1: Slide key for the breast cancer tissue array distribution.** This key shows the types of breast cancers on the breast cancer tissue array.
